# Caerin 1.1 and 1.9 inhibit glioblastoma growth associated with modulation of the ARHGAP26-β-catenin axis and enhancing intratumoral CD8^+^ T cell infiltration

**DOI:** 10.1371/journal.pone.0353182

**Published:** 2026-07-09

**Authors:** Furong Zhong, Jinyi Wu, Hongyin Wu, Yichen Wang, Fengyun Xiao, Bin Xu, Junjie Li, Yuandong Luo, Quanlan Fu, Xiaosong Liu, Tianfang Wang, Guoying Ni, Wei Zhang

**Affiliations:** 1 The First Affiliated Hospital, Clinical Medical School, Guangdong Pharmaceutical University, Guangzhou, China; 2 Zhongao Biomedical Technology (Guangdong) Co., Ltd., Zhongshan, China; 3 Cancer Research Institute, Foshan First People’s Hospital, Foshan, China; 4 Centre for Bioinnovation, University of the Sunshine Coast, Maroochydore, QLD, Australia; 5 School of Science, Technology and Engineering, University of the Sunshine Coast, Maroochydore, QLD, Australia; 6 Quality Assurance and Evaluation Center, Guangdong Pharmaceutical University, Guangzhou, China; Keio University School of Medicine Graduate School of Medicine: Keio Gijuku Daigaku Igakubu Daigakuin Igaku Kenkyuka, JAPAN

## Abstract

Glioblastoma (GBM) is an aggressive brain tumor with limited effective treatment options and poor patient outcomes. This study investigates the antitumor activity and underlying mechanisms of of host defense peptides caerin 1.1 (F1) and caerin 1.9 (F3) in glioblastoma models. F1/F3 treatment inhibited the proliferation of U87 cells and was associated with increased expression of ARHGAP26, suppression of β-catenin signaling pathway, and reduced the expression of downstream targets including MMP2, MMP7, and VEGFA. Cell death is primarily induced through apoptosis-related pathways, while pyroptosis-related and PI3K-related signaling showed more limited alterations. Notably, in immunodeficient NSG mice, F1/F3 altered the tumor immune microenvironment by promoting macrophage infiltration and M1-like polarization but did not significantly inhibit tumor growth. In contrast, in PBMC-humanized NSG mice, F1/F3 significantly suppressed U87 tumor growth and was associated with increased infiltration of macrophages and CD8^+^ T cells, together with reduced PD-L1 expression. These findings demonstrate that F1/F3 exerts both direct anti-tumor effects and immune-modulatory activities in glioblastoma models. The results support further investigation of caerin peptides as potential immunomodulatory therapeutics for glioblastoma.

## Introduction

Glioblastoma (GBM) is the most aggressive primary malignant brain tumor in adults [1]. It accounts for approximately 50% of all gliomas and 14.5% of all central nervous system tumors [2]. Despite advances in neurosurgical techniques and adjuvant therapy, the current standard of care, including maximal safe resection followed by radiotherapy and temozolomide (TMZ) extends median survival only modestly, typically to 14–18 months [3–5]. This poor prognosis arises from the diffuse infiltrative nature of GBM, extensive intratumoral heterogeneity, and a high degree of intrinsic and acquired resistance to cytotoxic therapy [6,7]. Tumor margins remain ill-defined and microinvasive cells persist in surrounding brain parenchyma even after aggressive resection, driving relapse and therapeutic failure [8–10].

In recent years, increasing attention has been directed toward the tumor microenvironment (TME) as a central determinant of GBM progression and therapy response. Unlike many peripheral solid tumors, GBM evolves within the unique immunological constraints of the central nervous system, leading to a highly immunosuppressive and metabolically hostile niche [11–13]. Its TME is characterized by abnormal and leaky vasculature, hypoxia, defective antigen presentation, excessive accumulation of M2-like tumor-associated macrophages (TAMs), myeloid-derived suppressor cells, and profoundly exhausted T-cell populations [13,14]. These features severely limit the recruitment, activation, and persistence of cytotoxic lymphocytes, rendering GBM one of the most refractory “cold tumors” to immunotherapy [15,16]. Consequently, immune checkpoint blockade, chimeric antigen receptor (CAR)-T cell therapy, and other T cell–based approaches have shown limited clinical benefit in GBM patients. Moreover, TMZ and radiotherapy can further suppress lymphocyte function, promoting immune escape and contributing to poor outcomes [17–19]. An effective therapeutic strategy for GBM will therefore require not only direct killing of tumor cells but also reprogramming of the immune microenvironment to enable robust antitumor immunity [20].

Host-defense peptides (HDPs) have recently emerged as a promising class of multifunctional therapeutic molecules capable of directly killing tumor cells and modulating immune responses. Among these, caerin 1.1 and caerin 1.9, amphipathic peptides originally identified from the skin secretions of Australian tree frogs, have demonstrated broad-spectrum antimicrobial activity and potent anticancer effects across a range of malignancies [21,22]. These peptides exert antitumor activity by disrupting cell membranes, altering mitochondrial function, modulating metabolic pathways, and influencing cytokine signaling. Our group and others have shown that caerin 1.1/1.9 (a 1:1 mixture) suppresses the growth of thyroid, breast, cervical, skin, and HPV-induced murine tumors [23–37]. Mechanistic studies have revealed that caerin 1.1/1.9 induces apoptosis via TNF-α signaling, reshapes macrophage heterogeneity, decreases immunosuppressive M2 macrophages, and enhances the efficacy of anti-PD-1 therapy by promoting an immunostimulatory TME [24,28,34,38].

In the context of glioblastoma, emerging evidence from our preliminary studies highlights several notable biological effects. Caerin 1.1/1.9 is capable of efficiently entering GBM cells, reducing mitochondrial membrane potential, suppressing glycolytic metabolism, and activating inflammatory pathways through downregulation of CHI3L1, which is an oncogenic factor associated with immune suppression and poor prognosis in GBM [39]. In addition to these mechanisms, our proteomic profiling of caerin-treated U87 cells identified a marked upregulation of ARHGAP26, a Rho GTPase–activating protein frequently downregulated in GBM and associated with enhanced tumor invasiveness [39,40]. ARHGAP26 has been reported to suppress tumor cell migration and invasion, in part through modulation of the β-catenin signaling pathway and downstream effectors involved in angiogenesis and extracellular matrix remodeling [41]. This observation suggests a previously unrecognized mechanism by which caerin peptides may influence cytoskeletal dynamics, transcriptional programs, and invasive behavior in GBM cells. Integrating ARHGAP26–β-catenin pathway regulation with metabolic and immunomodulatory effects highlights a potentially multifaceted role for caerin peptides in reshaping both tumor cell biology and the broader GBM microenvironment.

Despite these promising indications, the specific mechanisms through which caerin 1.1/1.9 remodels the GBM immune landscape and modulates key molecular pathways remain insufficiently understood. Therefore, this study aims to systematically dissect the mechanistic actions of caerin 1.1/1.9 in glioblastoma using complementary *in vitro* and *in vivo* approaches, with a particular focus on inflammatory signalling, metabolic regulation, ARHGAP26-β-catenin pathway involvement, and immune microenvironment reprogramming. These findings will provide a solid mechanistic foundation for advancing caerin-based therapeutic strategies as a novel immunomodulatory approach for GBM treatment.

## Materials and methods

### Cell lines

Human glioblastoma cell lines U87MG and U118MG were purchased from the Cell Bank of the Chinese Academy of Sciences. As per instructions, U87 cells were cultured in complete MEM medium, containing: MEM basal medium, penicillin-streptomycin, and 10% heat-inactivated fetal bovine serum. U118 cells were cultured in complete DMEM medium, containing: DMEM basal medium, penicillin-streptomycin, and 10% heat-inactivated fetal bovine serum. All cells were incubated at 37°C in a humidified atmosphere containing 5% CO_2_, as previously reported [39].

### Peptide synthesis

Caerin 1.1 (designated F1, sequence: GLLSVLGSVAKHVLPHVVPVIAEHL-NH_2_), Caerin 1.9 (designated F3, sequence: GLFGVLGSIAKHVLPHVVPVIAEKL-NH_2_), and a control peptide P3 (sequence: GTELPSPPSVWFAEFK-OH), which lacks cytotoxicity against various cancer cells, were synthesized by Shanghai Qiangyao Biological Technology Co., Ltd., China. Their purities, determined by reverse-phase high-performance liquid chromatography, were 99.47% (F1), 99.55% (F3), and 99.29% (P3), respectively. Caerin peptides and the P3 peptide were stored at 4°C until use.

### Cell viability assay

Cell viability was determined by the MTT method (Invitrogen) according to the manufacturer’s protocol. Briefly, 1.0 × 10⁴ U87 or U118 cells were seeded into 96-well plates and cultured overnight. The following day, cells were treated with various concentrations (range: 0–20 μg/mL) of F1, F3, or F1/F3 mixture (molar ratio 1:1) and incubated overnight at 37°C with 5% CO_2_. Then, 20 μL of 5 mg/mL MTT working solution was added to each well. After 4 h of incubation, 150 μL of DMSO was added, and the absorbance was measured at OD570 nm using a microplate reader (Thermo Fisher).

### Cell rescue assay

Cell viability post-inhibitor treatment was assessed using the CCK8 assay (Glpbio, GK10001) per the manufacturer’s instructions. Briefly, 1.0 × 10⁴ U87 or U118 cells were seeded into 96-well plates overnight. The next day, cells were pretreated with three inhibitors, including GSK-827 (HY-101872), necrosulfonamide (HY-100573), and liproxstatin-1 (HY-12726) (all purchased from Merck Millipore)—according to the instructions. After 3 h of incubation, cells were treated with various concentrations (range: 0–20 μg/mL) of the F1/F3 mixture and incubated overnight at 37°C with 5% CO_2_. On the third day, 10 μL of CCK8 reagent was added to each well, followed by an additional 2 hours of incubation. Absorbance was measured at OD570 nm using a microplate reader (Thermo Fisher).

### Lactate dehydrogenase (LDH) release assay

1 × 10⁴ U87 or U118 cells were seeded into 96-well plates and cultured under standard procedures. The next day, cells were treated as indicated. After treatment, culture supernatants were collected and centrifuged at 600 × g for 10 min. Supernatants from different experimental groups were transferred to a new 96-well plate, and LDH activity was detected using an LDH assay kit (Abcam, ab65393) according to the manufacturer’s protocol. LDH activity was calculated using the formula: (OD450 of sample - OD450 of low control)/ (OD450 of high control - OD450 of low control) × 100%, where the sample represents the supernatant from peptide-treated cells, the low control represents the supernatant from untreated cells, and the high control represents the supernatant from kit-provided lysed cells. All samples were tested in triplicate to ensure accuracy and reproducibility.

### Enzyme-linked immunosorbent assay (ELISA)

U87 and U118 cells were cultured to passage 2, seeded in 6-well plates at 1 × 10⁶ cells/well, and cultured for 18 hours. Cells were then stimulated with different concentrations (0, 2, 4, 6, 8, and 10 µg/mL) of the F1/F3 mixture for 1 h, after which the cell supernatants were collected. Levels of IL-1β, TNF-α, and IL-18 in the supernatants were measured using standard ELISA kits according to the manufacturers’ instructions. Human IL-1β/IL-1F2 Valukine™ ELISA Kit (VAL101) and Human IL-18 Valukine™ ELISA Kit (VAL131) were from R&D Systems. Human TNF-α ELISA Kit was purchased from Thermo Fisher.

### Protein extraction and western blotting

U87 cells (1 × 10⁶) were cultured in 6-well plates. Based on the IC50 value, cells were treated with 0 or 5 µg/mL of the peptides (set as blank control and experimental groups, respectively) overnight. Cells were lysed using RIPA lysis buffer (FD009, Fdbio science, China) supplemented with protease inhibitor (FD0100) and phosphatase inhibitor (FD1002). The lysates were scraped and briefly centrifuged. After determining the total protein concentration, samples were mixed with 5 × loading buffer (P0285, FdBioScience, China) and denatured by boiling at 100°C for 10 min. The samples were then separated by SDS-PAGE and transferred to PVDF membranes (FFP39, Beyotime). Membranes were blocked with Quick Block Western blocking buffer (P0018M-2, Beyotime) at room temperature for 1 h. Subsequently, the membranes were incubated overnight with primary antibodies against specific proteins of interest. Following this, membranes were incubated with HRP-conjugated secondary antibodies, and bands were visualized using an enhanced chemiluminescence substrate solution (T014-500, Beyotime, China). Immunoreactive bands were captured and analyzed using a MicroChemi 910 imaging system (Beijing Sage Creation Science Co., Ltd.) and Image J software (NIH, Bethesda, MD, USA). Antibodies used included: ARHGAP26 (ab180154, Abcam), β-catenin (8480, Cell Signaling Technology), MMP2 (ab92536, Abcam), MMP7 (ab205525, Abcam), VEGFA (2476, Cell Signaling Technology), Tubulin (ab7291, Abcam), GAPDH (EPR16891, Abcam). The original gel images were recorded in S1 File.

### Gene expression analysis

U87 cells (1 × 10⁶) were cultured in 6-well plates and stimulated with the F1/F3 peptide mixture for 1 h. Total RNA was extracted using Trizol reagent (Invitrogen), and RNA concentration and quality were assessed using a NanoDrop ND-2000 spectrophotometer (NanoDrop Technologies). RNA was then reverse transcribed into cDNA (Takara). Quantitative real-time PCR (qRT-PCR) analysis was performed using FastStart Universal SYBR Green Master (Roche, Shanghai, China) on a 7300 Real-Time PCR System (Applied Biosystems Inc., Foster City, CA, USA). After cDNA quality confirmation, gene expression profiling was conducted using human target qPCR arrays for Apoptosis Signaling Pathway Genes, Pyroptosis Signaling Pathway Genes, MAPK Signaling Pathway Genes, and PI3K-AKT Signaling Pathway Genes (all from Wcgene Biotech, Shanghai, China), according to the manufacturer’s protocols. Data were analyzed using Wcgene Biotech software (http://www.wcgene.com). Genes with a fold change greater than 2.0 or less than −2.0 were considered biologically significant. These genes are listed in S1 Table.

### Mice

All animal experiments were approved by the Animal Experiment Ethics Committee of the First Affiliated Hospital of Guangdong Pharmaceutical University (Approval No.: 00450216). Female NSG mice (NOD.CB17-Prkdcscid112rgtm1/Bcgen), aged 6–8 weeks, were purchased from Zhuhai Baise Tong Biotechnology Co., Ltd. and housed under specific pathogen-free (SPF) conditions in the institutional animal facility. Mice were maintained in a controlled environment (22°C, 75% humidity, 12-h light/dark cycle) with free access to standard rodent chow and water. Five mice were housed per cage. All procedures were conducted in accordance with institutional ethical guidelines and reported following the ARRIVE guidelines.

To minimize animal suffering and distress, mice were monitored daily for body weight, tumour burden, mobility, posture, grooming behaviour, and signs of respiratory distress or pain. Humane endpoint criteria were predefined and strictly implemented when any of the following conditions were observed: (1) tumour volume exceeded 2,000 mm³ or tumour diameter exceeded 1.5 cm; (2) body weight loss exceeded 15% within 24 h or remained above 10% for more than 3 consecutive days; or (3) mice showed impaired mobility, severe lethargy, or respiratory distress. Animals reaching humane endpoints were euthanized immediately and did not remain in the study beyond these criteria. No animals died unexpectedly prior to euthanasia.

For euthanasia, mice were first anesthetized by intraperitoneal injection of pentobarbital sodium (40 mg/kg) to reduce anxiety and discomfort, followed by carbon dioxide asphyxiation. Death was confirmed by the absence of cardiac activity for more than 5 min and the presence of fixed dilated pupils. All personnel involved in animal procedures had completed certified training in laboratory animal ethics, animal handling, identification of humane endpoints, anesthesia, and euthanasia procedures.

### Tumor model

A total of 21 female NSG mice (6 weeks old) were used to establish the glioblastoma xenograft model and were randomly assigned into three groups (n = 7 per group). All mice were purchased from Zhuhai Baise Tong Biotechnology Co., Ltd. and maintained under specific pathogen-free (SPF) conditions in accordance with institutional animal ethics guidelines. The experimental period lasted 21 days, including a 3-day acclimation period followed by an 18-day tumor establishment and treatment period.

After acclimation, U87 glioblastoma cells (5 × 10⁶ cells suspended in 200 µL PBS) were subcutaneously injected into the left flank of each mouse. Tumor formation was assessed 3 days after inoculation. Following successful tumor engraftment, mice received the indicated treatments for 14 consecutive days. During the experimental period, mice were monitored daily for general health condition, body weight, mobility, grooming behavior, and signs of pain or distress. Tumor dimensions were measured every two days using digital calipers, and tumor volume was calculated using the formula: volume = length × (width²/ 2).

At the experimental endpoint (day 21), mice were anesthetized according to the protocol described above, and deep anesthesia was confirmed prior to euthanasia by cervical dislocation. Tumors were immediately excised, photographed, weighed, and measured for final tumor volume analysis. All animals were euthanized at the predetermined experimental endpoint, and no animals died unexpectedly during the study. Humane endpoint criteria were applied throughout the experiment in accordance with approved ethical protocols. All procedures were approved by the Animal Ethics Committee of the First Affiliated Hospital of Guangdong Pharmaceutical University.

### Construction of PBMC humanized mouse model

Peripheral venous blood was collected from healthy adult donors following written informed consent. Peripheral blood mononuclear cells (PBMCs) were isolated according to the manufacturer’s instructions and resuspended in sterile PBS for transplantation. All procedures involving human blood samples were approved by the Institutional Review Board (Ethics Committee) of the First Affiliated Hospital of Guangdong Pharmaceutical University (Ethics Approval No.: [2025] IIT (45)). Donor recruitment and blood collection were conducted between 25/07/2025 and 20/02/2026 in accordance with approved ethical guidelines.

A total of seven female NSG mice (6 weeks old) were used to establish the PBMC-humanized glioblastoma model. Mice were maintained under specific pathogen-free (SPF) conditions, and the total experimental duration was 30 days, including a 3-day acclimation period followed by a 27-day intervention and observation period. After acclimation, mice received 5 × 10⁶ human PBMCs suspended in PBS via tail vein injection. A second PBMC injection was performed 9 days later to enhance human immune cell engraftment. One mouse was randomly selected for flow cytometric analysis to confirm human immune cell reconstitution by assessing the proportion of hu-CD45 + immune cell subsets. The remaining six mice were randomly assigned into two groups (n = 3 per group).

On day 13, U87 glioblastoma cells (5 × 10⁶ cells in 200 µL PBS) were subcutaneously injected into the lateral flank of each mouse. Following successful tumor establishment, mice received the indicated treatments for 14 consecutive days. During the study, mice were monitored daily for body weight, tumor burden, mobility, grooming behaviour, and signs of pain or distress. Tumor size was measured every two days using digital calipers, and tumor volume was calculated using the formula: volume = length × (width²/ 2).

At the experimental endpoint (day 30), mice were anesthetized according to the protocol described above, and deep anesthesia was confirmed prior to euthanasia by cervical dislocation. Tumors were immediately excised, weighed, and measured for tumor volume analysis. All animals were euthanized at the predetermined experimental endpoint, and no animals died unexpectedly during the study. Humane endpoint criteria were strictly implemented throughout the experiment in accordance with approved animal ethics protocols.

### Peptide treatment of tumors

Three days after tumor cell inoculation (tumor diameter: 3–5 mm), the experimental group:mice received daily intratumoral injections of 100 µL PBS containing 30 µg of the F1/F3 mixture for 14 consecutive days. The blank control group mice received daily intratumoral injections of 100 µL of PBS for 14 consecutive days; and the negative control group mice received daily intratumoral injections of 100 µL of PBS containing 30 µg of the P3 peptide for 14 consecutive days.

### Hematoxylin and eosin (H&E) staining

Tissue sections were sequentially immersed in xylene I and xylene II (15 min each) for deparaffinization, followed by rehydration through a graded ethanol series: 100% ethanol I and II (5 minutes each), then 95%, 90%, 80%, 70%, and 50% ethanol (5 min each). After rinsing three times with distilled deionized water (ddH₂O), sections were stained with Harris hematoxylin for 20 min and rinsed under running water for 30 min for nuclear bluing (care was taken to avoid direct water flow onto the sections). After three washes with ddH₂O, dehydration was performed sequentially with 50%, 70%, 80%, and 90% ethanol (2 min each). Cytoplasmic counterstaining was performed using Eosin Y solution for 10 s. Final dehydration steps were: 95% ethanol I and II (2 min each), 95% ethanol II (2 min), 100% ethanol I (5 min), 100% ethanol II (5 min), followed by clearing in xylene I and II (5 min each). Sections were permanently mounted with neutral resin, and images were acquired using a ZEISS AX10 optical microscope.

### Multiplex immunofluorescence sample pretreatment and staining

Formalin-fixed, paraffin-embedded tissue blocks were sectioned into 3 μm thick slices and floated in a 40°C water bath. Once fully flattened, sections were mounted onto glass slides. Mounted sections were dried overnight in a 42°C oven to ensure tissue adherence, or alternatively baked at 65°C for 1 h. Deparaffinization was performed by vertically incubating slides in xylene for 3 hours. Rehydration was carried out through a graded ethanol series (absolute ethanol, 95% ethanol, 70% ethanol, and 50% ethanol, 20 min each) and pure water. Antigen retrieval was performed using alkaline retrieval solution at 95°C for 20 min, followed by natural cooling for 40 min to room temperature. Retrieved slides were stored in PBS buffer at 4°C. To eliminate autofluorescence, slides were immersed in 3% hydrogen peroxide solution and irradiated using a 50 W LED light source in two sessions (45 min each, with solution change between sessions). After two washes with PBS, slides were stored in PBS buffer. Permeabilization was performed using PBS containing 0.5% Triton X-100 at room temperature for 15 min. After two PBS washes, slides were stored. Staining was finally performed using the multiplex immunofluorescence kit compatible with the MGISEQ-2000RS FluoXpert system, and stained images were acquired and observed via the FluoXpert staining software interface. The raw data were recorded in S2 Table.

### Flow cytometry for detection of cell surface markers

To obtain single cells, U87 tumor tissues implanted in the flanks of NSG mice were dissected and homogenized using a Tumor Dissociation Kit (Miltenyi Biotec, Bergisch Gladbach, Germany). Subsequently, single cells were stained with different antibodies as described below. Live cells were analyzed using a flow cytometer (BD Biosciences, San Jose, CA, USA, Model FACS Aria II), and data were analyzed using Flow Jo v10.0 software (Tree Star Inc., Ashland, OR, USA). Antibodies used for flow cytometry are listed in S3 Table.

### Statistical analysis

Flow cytometry data were analyzed using FlowJo v10 software (Tree Star Inc., Ashland, OR, USA). Statistical analyses and graph generation were performed using GraphPad Prism 9 software (GraphPad Software, San Diego, CA, USA). Data are presented as mean ± standard error of the mean (SEM) unless otherwise indicated. All *in vitro* experiments were performed with at least three independent biological replicates unless otherwise stated.

For comparisons between two groups, unpaired two-tailed Student’s t-tests were used. For comparisons involving more than two groups, one-way analysis of variance (ANOVA) followed by appropriate post hoc multiple-comparison tests was performed. Tumor growth curves were analyzed using two-way ANOVA where appropriate. A *P* value < 0.05 was considered statistically significant. Statistical significance is indicated in the figures as follows: *P < 0.05, **P < 0.01, ***P < 0.001, and ****P < 0.0001.

## Results

### F1/F3 induces atypical pyroptosis-associated features in U87 and U118 glioblastoma cells

**Fig 1:**
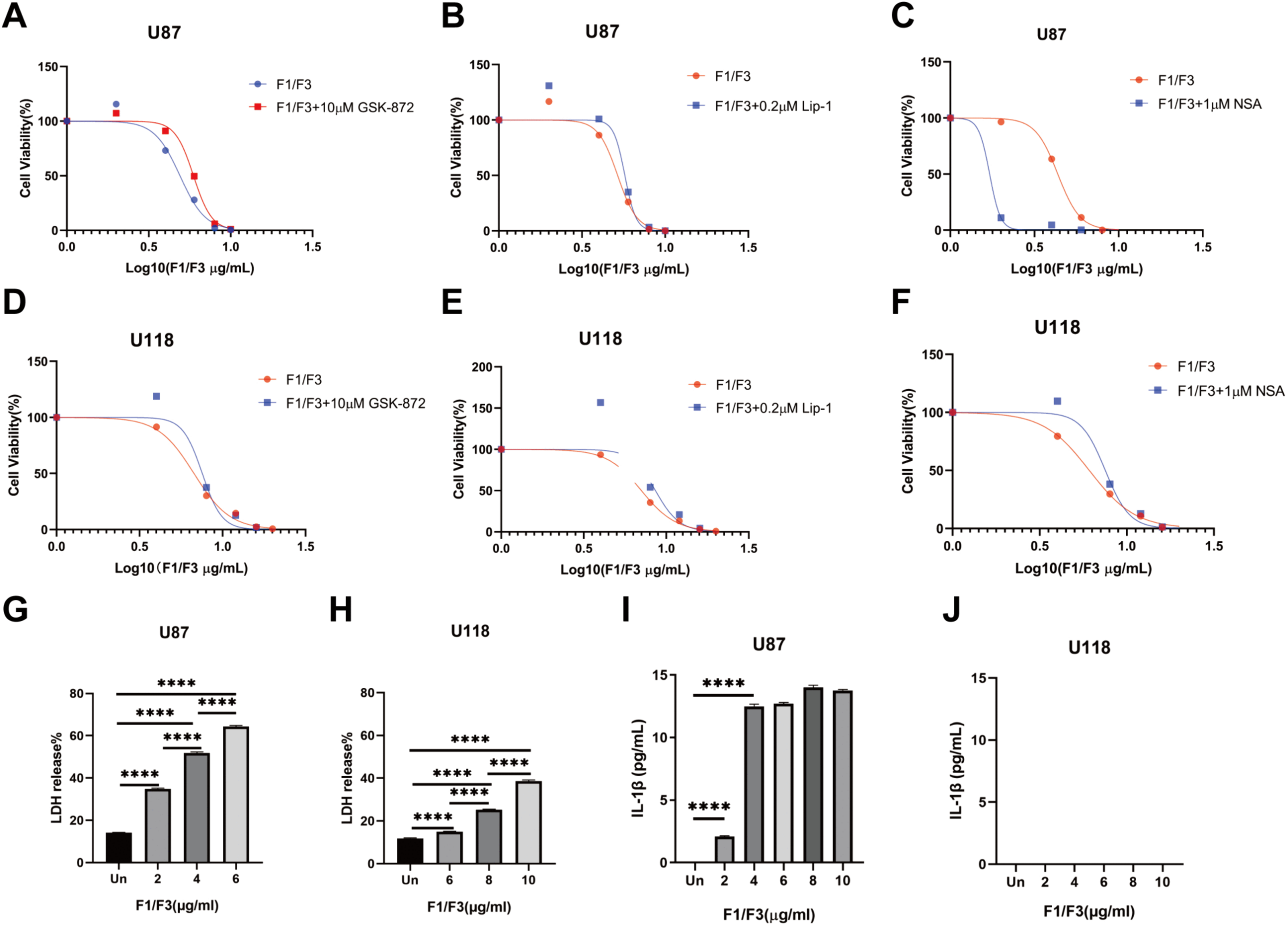
F1/F3-induced U87 and U118 cell death is independent of ferroptosis or necroptosis pathways. **(A–C)** Cell viability of U87 cells treated with increasing concentrations of the F1/F3 peptide mixture in the presence or absence of **(A)** GSK-872 (necroptosis inhibitor), **(B)** Liproxstatin-1 (ferroptosis inhibitor), or **(C)** Necrosulfonamide (NSA; necroptosis inhibitor). (D–F) Corresponding cell viability analyses in U118 cells treated with F1/F3 in combination with the indicated inhibitors. Cell viability was determined by CCK8 assay. **(G–H)** Lactate dehydrogenase (LDH) release from **(G)** U87 and **(H)** U118 cells following 1 h treatment with the indicated concentrations of F1/F3, expressed as a percentage of maximal LDH release. **(I–J)** IL-1β secretion in **(I)** U87 and **(J)** U118 cells after 24 h treatment with F1/F3, measured by ELISA. Data are presented as mean ± SEM from at least three independent experiments. Statistical significance was analyzed using one-way ANOVA or unpaired Student’s t-test where appropriate. *P < 0.05, **P < 0.01, ***P < 0.001, ****P < 0.0001.

Previous studies showed that F1/F3 inhibits the proliferation of U87 and U118 glioblastoma cells. In line with these findings, our MTT assays confirmed a dose-dependent reduction in cell viability, with IC_50_ values of 4.614 µg/mL for U87 cells and 8.026 µg/mL for U118 cells (S1A and S1B Fig). To determine the mode of cell death induced by F1/F3, cells were pretreated with inhibitors targeting distinct pathways, including liproxstatin-1 (ferroptosis inhibitor), necrosulfonamide and GSK-872 (necroptosis inhibitors) None of these inhibitors restored cell viability following F1/F3 treatment (Fig 1A to 1F), indicating that F1/F3-induced cytotoxicity is not mediated by ferroptosis or necroptosis. Interestingly, NSA pretreatment further enhanced F1/F3 cytotoxicity in U87 cells (Fig 1C), suggesting possible crosstalk between the F1/F3-activated death pathway and necroptosis-related signaling.

Given our previous observation that F1/F3 induces caspase-3/GSDME-dependent pyroptosis in HeLa cells, we next examined whether a similar mechanism occurs in glioblastoma cells. Lactate dehydrogenase (LDH) release, a hallmark of membrane rupture during pyroptosis, was significantly increased following F1/F3 treatment in both cell lines. In U87 cells, F1/F3 at 0, 2, 4, and 6 µg/mL induced LDH release of 14.14 ± 0.34%, 34.91 ± 0.66%, 51.85 ± 1.10%, and 64.31 ± 1.12%, respectively (Fig 1G and 1H**)**. In U118 cells, LDH release increased 11.84 ± 0.56% (control) to 15.03 ± 0.32%, 25.29 ± 0.50%, and 38.59 ± 1.25% at 6, 8, and 10 µg/mL F1/F3, respectively. These results demonstrate substantial plasma membrane damage induced by F1/F3.

To further characterize the nature of this death pathway, we measured extracellular IL-1β, IL-18, TNF-α cytokines commonly released during canonical pyroptosis. In U87 cells, only trace IL-1β was detected after F1/F3 treatment, and IL-18 and TNF-α remained below detection limits (Fig 1I and S2A-B Fig). None of the three cytokines were detected in U118 cells (Fig 1J, S2C-D Fig). Despite the marked LDH release and membrane disruption observed in both cell lines, the absence of substantial inflammatory cytokine secretion suggests that F1/F3 does not induce classical canonical pyroptosis in glioblastoma cells. Instead, these findings support the presence of pyroptosis-associated or membrane lytic features accompanied predominantly by non-canonical inflammatory responses.

### F1/F3 modulates ARHGAP26–β-catenin axis and apoptosis-related signaling in U87 glioblastoma cells

**Fig 2:**
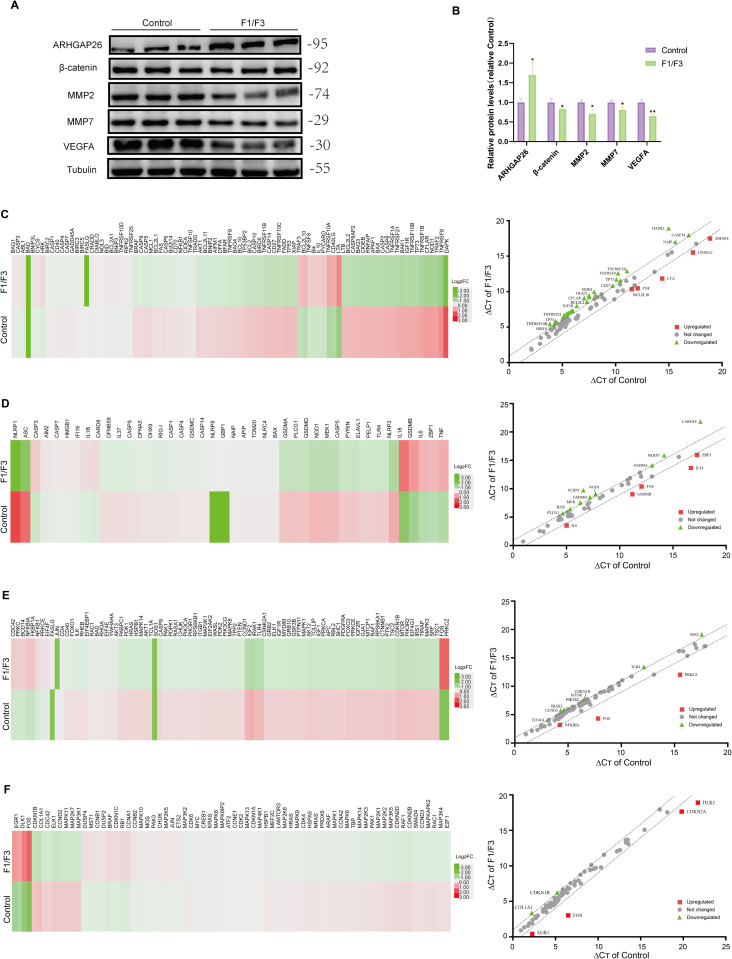
F1/F3 modulates ARHGAP26–β-catenin signaling and alters cell death–related gene expression in U87 glioblastoma cells. **(A)** Western blot analysis of ARHGAP26, β-catenin, MMP2, MMP7, VEGFA, and Bcl-2 in U87 cells treated with 5 µg/mL F1/F3 for 1 h. Tubulin and GAPDH were used as loading controls. (see S1 File for original gel images) **(B)** Densitometric quantification of protein expression levels normalized to Tubulin or GAPDH. **(C-F)** Quantitative PCR array analysis of differentially expressed genes in U87 cells treated with 5 μg/mL F1/F3 for 1 h, related to **(C)** apoptosis, **(D)** pyroptosis-related signaling, **(E)** PI3K-AKT signaling, and **(F)** MAPK signaling pathways. Only genes with fold change > 2 are presented. Data shown in (**A**–**B**) are representative of at least three independent experiments..

To investigate whether F1/F3 regulates the ARHGAP26–β-catenin axis, we examined the protein expression of ARHGAP26, β-catenin, and downstream effector molecules (VEGFA, MMP2, and MMP7) by western blotting. F1/F3 treatment markedly increased ARHGAP26 protein levels in U87 cells, while β-catenin and its downstream targets were substantially downregulated (Fig 2A and 2B). These data support the hypothesis that F1/F3 activates ARHGAP26 while suppressing β-catenin–mediated transcriptional programs associated with angiogenesis and invasiveness.

To further delineate the molecular pathways involved in the response to F1/F3, transcriptomic profiling was performed. Genes within the apoptosis pathway showed the most pronounced changes, with 35 differentially expressed genes. Both pro-apoptotic and anti-apoptotic regulators, as well as death receptor–related genes, were significantly altered, indicating that apoptosis is a major pathway affected by F1/F3 (Fig 2C).

Pyroptosis-related genes also exhibited notable changes, with 14 differentially expressed genes. Among these, pro-inflammatory cytokines such as IL18 and IL6 were upregulated, whereas key pore-forming effector molecules including GSDMA and GSDMD were downregulated (Fig 2D). This pattern aligns with our functional assays showing the presence of atypical pyroptosis-associated features, suggesting that pyroptotic signalling contributes to but is not the primary driver of the cytotoxic effects of F1/F3.

Additionally, 11 genes within the PI3K pathway were differentially expressed, including members of the PI3K and AKT families (Fig 2E). These alterations imply that PI3K/AKT signalling may participate in the cellular response to F1/F3 and could cooperate with apoptosis-related mechanisms. In contrast, only six genes within the MAPK pathway showed significant changes (Fig 2F), indicating that MAPK signalling likely plays a limited role in F1/F3-mediated inhibition of U87 cell proliferation.

Collectively, these findings demonstrate that F1/F3 upregulates ARHGAP26 and suppresses β-catenin signalling while inducing broad transcriptional changes primarily dominated by apoptosis-related genes. Pyroptosis-associated and PI3K-related pathways appear to contribute as secondary or supportive mechanisms. These results suggest that apoptosis represents the principal pathway through which F1/F3 exerts its anti-tumor effects in U87 glioblastoma cells.

### F1/F3 promotes macrophage infiltration and M1-like polarization in U87 tumors

**Fig 3:**
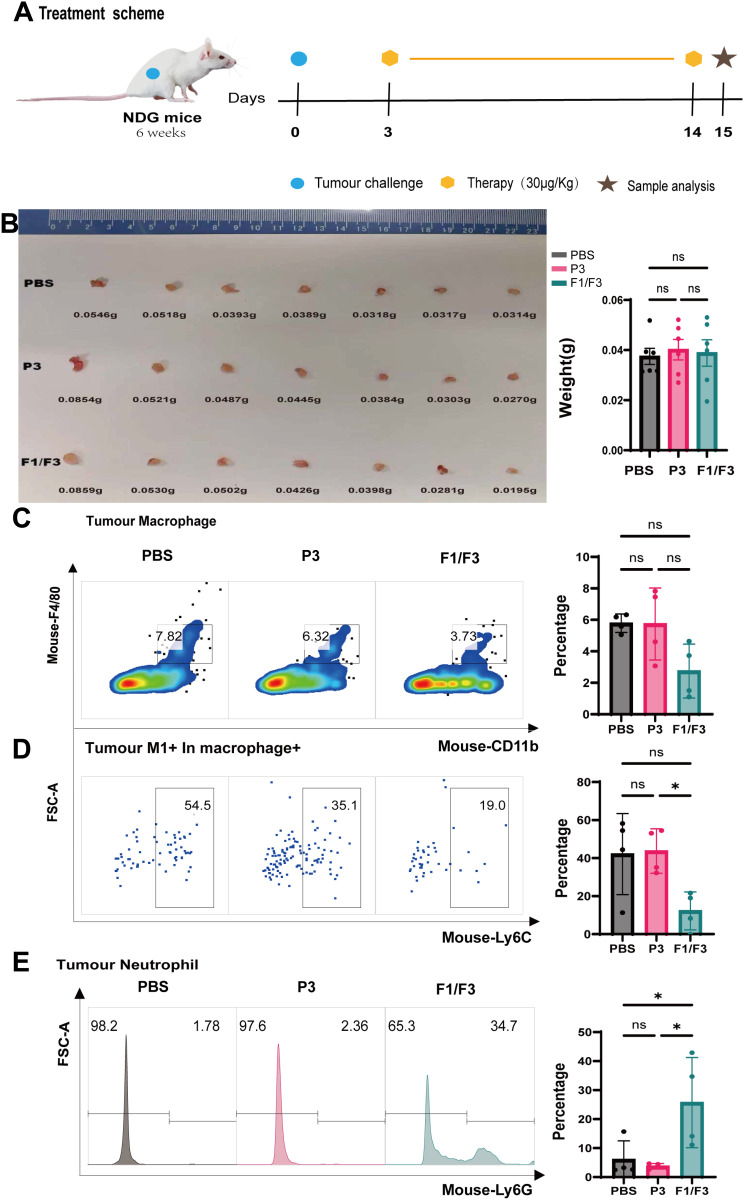
F1/F3 promotes macrophage recruitment and M1 polarization in U87 tumors in NSG mice. **(A)** Schematic illustration of the treatment protocol in NSG mice bearing subcutaneous U87 tumors. Mice received daily intratumoral injections of PBS, control peptide P3, or F1/F3 (30 μg/mouse) for 14 consecutive days. **(B)** Tumor weights at the experimental endpoint (day 15) in mice treated with PBS, P3, or F1/F3 (n = 7 per group). Flow cytometric analysis of immune cell populations within the TME of PBS-, P3-, and F1/F3-treated U87 tumor-bearing mice, including **(C)** macrophages cells (CD45^+^CD11b^+^F4/80^+^), **(D)** M1 cells (CD45^+^CD11b^+^F4/80^+^Ly6C^+^). **(E)** neutrophil cells (CD45.2^+^CD11b^+^Ly6G^+^). Data are presented as mean ± SD. Results are representative of two independent experiments. Statistical significance was determined using one-way ANOVA with appropriate multiple-comparison testing. (ns, not significant, **: *P* < 0.01, ***: *P* < 0.001, ****: *P* < 0.0001.).

To evaluate the impact of F1/F3 on tumor growth and the immune microenvironment *in vivo*, a xenograft model was established by subcutaneously transplanting U87 cells into the flanks of NSG mice. Once tumors formed, mice received intratumoral F1/F3 injection for two weeks, and tumors were collected on day 15 (Fig 3A). Consistent with our previous findings in the HeLa nude mouse model, F1/F3 did not significantly alter tumor size compared with PBS or the control peptide P3 (Fig 3B), indicating that its antitumor efficacy requires an intact immune system and is not readily observable in immunodeficient mouse models.

We next examined immune cell infiltration in the tumors and spleens by flow cytometry. In U87 tumors, F1/F3 treatment significantly increased the proportion of neutrophils (Fig 3E) but did not markedly change total leukocyte numbers, macrophage frequency, or M1-type macrophage abundance (Fig 3C and 3D). A slight decreasing trend in tumor-associated macrophages was observed. In contrast, splenic immune profiling revealed no change in total leukocyte numbers but a significant increase in macrophages, neutrophils, and M1-like macrophages following F1/F3 administration (S3A and S3C Fig). These findings suggest that although overall immune cell abundance in U87 tumors remained relatively stable, F1/F3 altered inflammatory cell composition and promoted systemic activation of myeloid populations.

**Fig 4:**
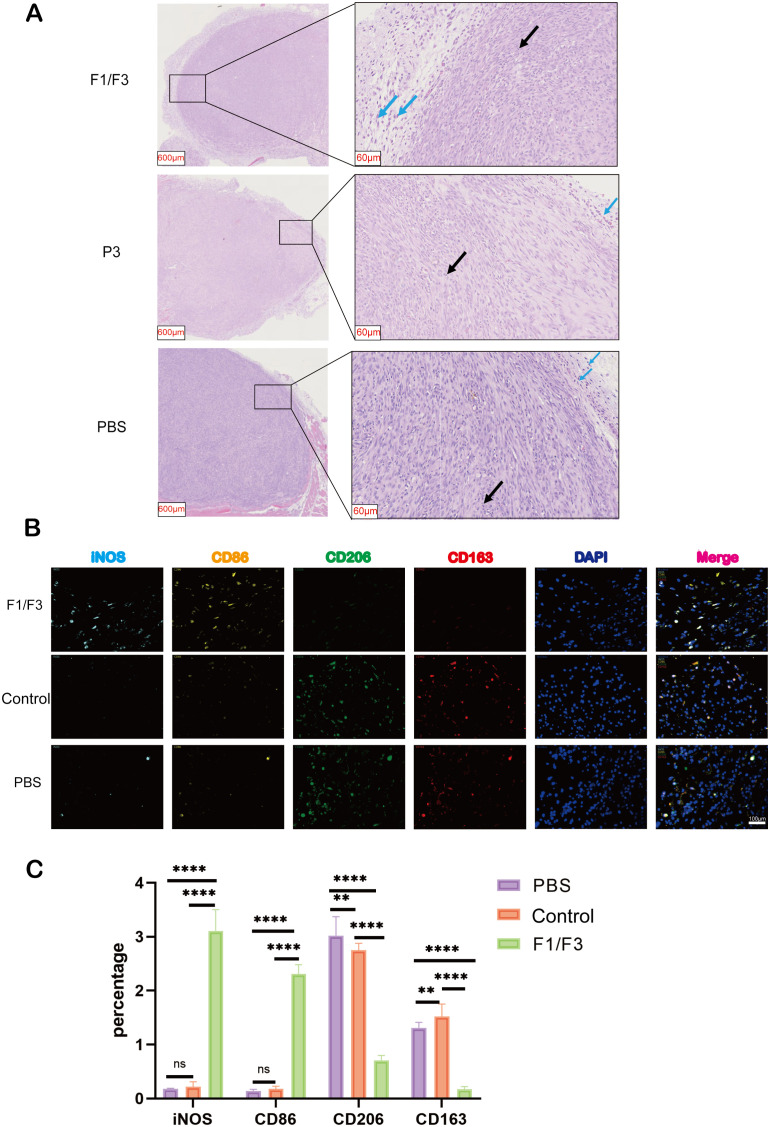
F1/F3 alters tumor histology and induces M1 polarization of tumor-associated macrophages. **(A)** Representative hematoxylin and eosin (H&E) stained tumor sections from PBS-, P3-, and F1/F3-treated U87 tumor-bearing NSG mice. Left panels: scale bar = 600 μm; right panels: scale bar = 60 μm. Black arrows indicate infiltrating immune cells at tumor margins. **(B)** Representative multiplex immunofluorescence staining of M1 markers (iNOS, CD86) and M2-associated markers (CD206, CD163) in tumor tissues. Nuclei were counterstained with DAPI (see S). **(C)** Quantification of the percentage of cells positive for iNOS, CD86, CD206, and CD163 in the tumor sections. Data are presented as mean ± SD. Statistical significance was determined using one-way ANOVA with appropriate multiple-comparison testing. ns, not significant; * *P* < 0.05; ** *P* < 0.01; *** *P* < 0.001; **** *P* < 0.0001.

Histological analysis provided further insight into local immune responses. H&E staining showed densely packed glioma cells with high nuclear-to-cytoplasmic ratios across all groups; however, tumors from F1/F3-treated mice exhibited noticeably greater infiltration of lymphocytes and macrophages at the tumor margin compared with PBS or P3 controls (Fig 4A). This increased peri-tumoral infiltration is consistent with an F1/F3-induced inflammatory response.

To more precisely characterize macrophage phenotypes, multiplex immunofluorescence staining was performed on tumor sections. Macrophages were predominantly localized at tumor margins in all groups, but F1/F3 treatment markedly altered their polarization status. In the F1/F3 group, expression of M1-associated markers iNOS and CD86 was significantly elevated, whereas immunosuppressive M2 markers CD206 and CD163 were strongly reduced (Fig 4B and 4C). In contrast, PBS and P3 groups retained a predominantly M2-like macrophage profile. These results demonstrate that F1/F3 not only enhances macrophage infiltration around U87 tumors but also reprograms macrophages toward a pro-inflammatory, antitumor M1 phenotype. These data showed that F1/F3 modulated the TME by recruiting macrophages to the tumor margin and shifting macrophage polarization away from an immunosuppressive M2 phenotype toward an inflammatory M1 phenotype, consistent with its immunomodulatory effects observed in B16 and TC-1 tumor models.

### F1/F3 suppresses U87 tumor growth in humanized NSG mice by enhancing CD8^+^ T cell infiltration and reducing PD-L1 expression

**Fig 5:**
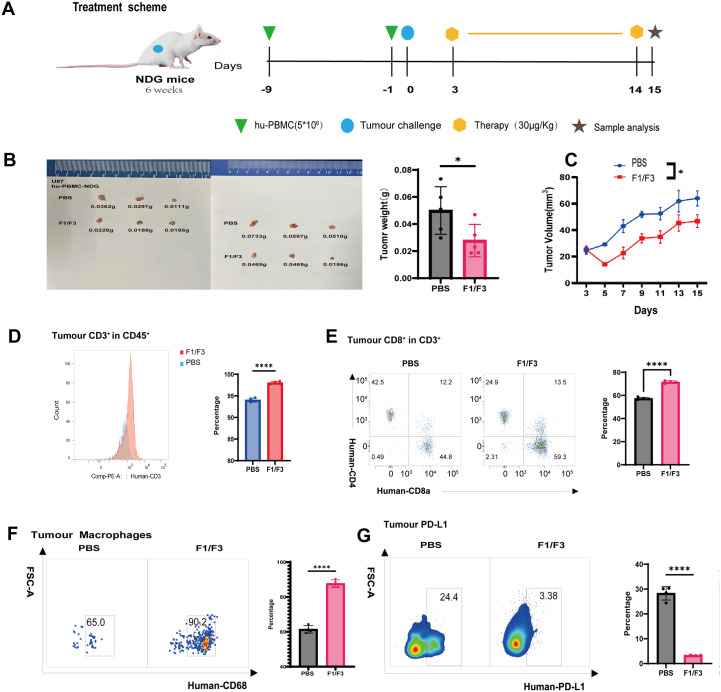
F1/F3 inhibits U87 tumor growth in humanized NSG mice by enhancing CD8^+^ T cell infiltration and reducing PD-L1 expression. **(A)** Schematic diagram of the humanized NSG mouse model and treatment schedule. PBMCs (5 × 10^6^ cells) were injected intravenously on days 0 and 9. U87 tumor cells were inoculated subcutaneously on day 3, followed by daily intratumoral F1/F3 injections from day 6 to day 19. **(B)** Tumor weight data (n = 6). **(C)** Tumor growth curve. Flow cytometric analysis in the TME of PBS and F1/F3-treated U87 tumor-bearing mice, including **(D)** Human T cells (Mouse CD45.2^-^ human CD45^+^CD3e^+^). **(E)** Human CD8^+^ T cells (Mouse CD45.2^-^ human CD45^+^CD3e^+^CD8^+^). **(F)** Human Macrophages (Mouse CD45.2^-^humanCD45^+^CD11b^+^CD68^+^). **(G)** Human PD-L1 (Mouse CD45.2^-^ human CD45.2^+^ Lineage-PD-L1^+^). **(A-G)** represent single independent experiments, each repeated once. Data are expressed as mean ± SD. (ns: ns, not significant, *: *P* < 0.05, ****: *P* < 0.0001.).

To further determine whether the antitumor activity of F1/F3 requires an intact immune system, we established a humanized microenvironment by intravenously transferring human PBMCs into NSG mice (Fig 5A). Successful reconstitution was confirmed by flow cytometry and histological analysis (S4A Fig), U87 glioma xenograft tumors were then generated in these mice, followed by intratumoral F1/F3 administration. Unlike in non-humanized mice, F1/F3 treatment significantly reduced tumor weight and volume compared with the PBS group (Fig 5B and 5C), demonstrating that F1/F3 effectively inhibits glioma growth when functional human immune components are present.

We next assessed human immune cell infiltration within the tumors. Flow cytometry revealed a marked increase in the proportion of total human T cells (FVS510^+^CD45^+^CD3e^+^) in the F1/F3 group relative to PBS controls (Fig 5D). Notably, human CD8^+^ cytotoxic T cells increased significantly, indicating enhanced recruitment or expansion of effector T-cell populations (Fig 5E). Human macrophage infiltration was also elevated following F1/F3 treatment (Fig 5F), consistent with the macrophage-modulating effects observed in non-humanized models. Importantly, F1/F3 treatment significantly downregulated PD-L1 expression on tumor cells (Fig 5G), suggesting reduced immune checkpoint engagement and alleviation of tumor-mediated immunosuppression.

Collectively, these data demonstrate that F1/F3 reshapes the glioma immune microenvironment in humanized mice by simultaneously enhancing CD8 ⁺ T-cell infiltration and reducing PD-L1 expression on tumor cells. This dual action promotes more effective antitumor immunity and provides compelling evidence that the therapeutic effect of F1/F3 is dependent on the presence of functional immune components. These findings further support the potential of F1/F3 as a candidate for combination immunotherapy strategies targeting GBM.

## Discussion

This study expands our previous work demonstrating that the host-defense caerin 1.1 (F1) and caerin 1.9 (F3) exert potent anticancer effects against glioblastoma. Our earlier work showed that F1/F3 enters glioblastoma cells, disrupts mitochondrial membrane potential, suppresses glycolysis, downregulates CHI3L1, and inhibits glioblastoma cell proliferation and migration [39]. In the present study, we further investigated the mechanisms underlying F1/F3-mediated cytotoxicity and evaluated, for the first time, its effects on tumor growth and immune cell infiltration in both immunodeficient and PBMC-humanized U87 xenograft models.

Host-defense peptides frequently induce multiple forms of cellular stress through interactions with biological membranes [42]. Consistent with this concept, F1/F3-induced cytotoxicity in glioblastoma cells was not prevented by inhibitors of ferroptosis or necroptosis, suggesting that neither pathway represents the dominant mechanism of cell death. Interestingly, necrosulfonamide enhanced F1/F3-mediated cytotoxicity in U87 cells but not U118 cells, highlighting the influence of glioblastoma heterogeneity on cell death responses. These findings suggest that F1/F3 initiates a complex stress response that may engage multiple cell death pathways depending on cellular context.

Our data indicate that apoptosis is likely the predominant mechanism underlying F1/F3-induced glioblastoma cell death. PCR array analyses revealed extensive regulation of apoptosis-related genes, whereas alterations in pyroptosis-related and PI3K-related pathways were less pronounced. Previous studies from our group have demonstrated that F1/F3 can induce caspase-dependent cell death in other tumor models [43], supporting the possibility that similar mechanisms contribute to the observed effects in glioblastoma. However, direct functional validation of apoptosis, such as Annexin V/PI staining, caspase activation assays, or caspase inhibition studies, was not performed in the present study. Therefore, our findings should be interpreted as being consistent with apoptosis as the dominant mechanism rather than providing definitive proof.

Although several pyroptosis-related genes were differentially expressed following F1/F3 treatment, the evidence did not support canonical inflammasome-mediated pyroptosis as a major mechanism. In both U87 and U118 cells, IL-1β and IL-18 secretion was minimal or absent, and key canonical pyroptosis effectors, including GSDMD, were downregulated. These findings suggest that F1/F3 treatment is associated with certain pyroptosis-related signaling changes but does not induce classical pyroptosis in glioblastoma cells. Future studies examining inflammasome activation, gasdermin cleavage, and caspase-dependent signaling will be necessary to determine whether pyroptosis-related pathways contribute to F1/F3-mediated cytotoxicity.

Integrating previous proteomic data [39] with validation experiments in this study, we identified ARHGAP26 as a candidate pathway associated with the antitumor effects of F1/F3. ARHGAP26 is a Rho GTPase-activating protein that terminates RhoA signaling, regulating cytoskeletal remodeling, cell migration, and apoptosis [44,45]. ARHGAP26 is markedly downregulated in glioblastoma and other malignancies [40,46–48], supporting a potential tumor-suppressive role. In this study, F1/F3 treatment upregulated ARHGAP26 expression and was accompanied by suppression of β-catenin and downstream targets including MMP2, MMP7, and VEGFA, which are known mediators of drivers of glioblastoma proliferation, angiogenesis, and invasion [49–52]. Similar observations have been reported in ovarian cancer, where ARHGAP26 inhibits β-catenin signaling and reduces metastatic capacity [41]. The upregulation of ARHGAP26 and inhibition of β-catenin signaling observed in this study integrate well with our previous metabolic findings [25]. Although these findings support an association between F1/F3 treatment and modulation of the ARHGAP26–β-catenin axis, direct mechanistic validation through ARHGAP26 knockdown or overexpression studies was not performed. Therefore, the present study identifies ARHGAP26 as a promising candidate mediator warranting further investigation rather than establishing a definitive causal mechanism.

The TME plays a decisive role in glioblastoma progression and resistance to therapy [53–56]. Consistent with our previous observations [28], F1/F3 altered the composition of immune cells within the TME of immunodeficient NSG mice. Specifically, F1/F3 increased macrophage infiltration, promoted M1-like macrophage polarization, and increased neutrophil abundance within tumors. However, despite these immunological changes, no significant inhibition of tumor growth was observed in immunodeficient mice, suggesting that innate immune modulation alone may be insufficient for robust antitumor activity.

In contrast, significant tumor suppression was observed in PBMC-humanized NSG mice. F1/F3 treatment was associated with increased infiltration of human CD8 ⁺ T cells and macrophages, together with reduced PD-L1 expression within tumors. These findings suggest that human immune cells contribute substantially to the antitumor activity of F1/F3. However, the increased presence of CD8 ⁺ T cells should not be interpreted as direct evidence of enhanced cytotoxic function, as activation status, cytokine production, exhaustion markers, and killing capacity were not evaluated in the current study. Additional functional studies will be required to determine whether F1/F3 enhances CD8 ⁺ T-cell effector activity in glioblastoma.

An important limitation of this study relates to the PBMC-humanized NSG model itself. Human T cells reconstituted in this system are predominantly naïve and do not fully recapitulate the chronically stimulated and exhausted phenotype characteristic of tumor-infiltrating lymphocytes in human glioblastoma. Consequently, the model cannot fully reproduce the highly immunosuppressive immune landscape observed in patients. Furthermore, the relatively small sample size used in the humanized mouse experiments limits statistical power and warrants cautious interpretation of the immune findings. Therefore, the results should be considered preliminary evidence that F1/F3 can modulate human immune cell infiltration within glioblastoma tumors. Future studies employing more advanced humanized models, larger cohorts, and functional immune analyses will be important for validating the translational relevance of these observations.

Overall, our findings demonstrate that F1/F3 exerts antitumor activity through a combination of direct tumor-cell effects and modulation of the tumor immune microenvironment. The data support apoptosis-associated mechanisms as the primary contributor to glioblastoma cell death and identify modulation of the ARHGAP26–β-catenin pathway as a potential component of the response to F1/F3 treatment. In addition, F1/F3 promotes immune cell infiltration and reduces PD-L1 expression in humanized glioblastoma models. Together, these findings support further investigation of caerin peptides as potential immunomodulatory agents for glioblastoma therapy, particularly in combination with existing immunotherapeutic approaches.

## Conclusions

In summary, F1/F3 exhibited significant antitumor activity against glioblastoma through a combination of direct tumor-cell effects and modulation of the tumor immune microenvironment. *In vitro*, F1/F3 treatment was associated with modulation of the ARHGAP26–β-catenin signaling axis, suppression of β-catenin downstream targets, and broad transcriptional changes predominantly involving apoptosis-related pathways. *In vivo*, F1/F3 altered the composition of immune cells within the tumor microenvironment, promoting macrophage infiltration and M1-like polarization in non-humanized mice. In PBMC-humanized NSG mice, F1/F3 treatment was associated with increased infiltration of human CD8 ⁺ T cells and macrophages, reduced PD-L1 expression, and significant inhibition of U87 tumor growth. The present study identifies F1/F3 as a promising immunomodulatory peptide with both direct antitumor and immune-regulatory properties. However, several limitations warrant further investigation, including the lack of direct functional validation of the ARHGAP26 pathway, limited characterization of T-cell effector function, and the inherent constraints of the PBMC-humanized mouse model, to support its future translational development for glioblastoma therapy.

## Supporting information

S1 Table Raw CCK-8 assay data for U87 and U118 cell viability experiments. (XLSX)

S2 Table Raw multiplex immunofluorescence quantification data for U87 tumor sections. (XLSX)

S3 Table Human Apoptosis-, Pyroptosis-, PI3K-AKT-, and MAPK Signaling-Related Genes Included in the qPCR Arrays. (DOCX)

S4 Table Antibodies used for flow cytometry analysis. (DOCX)

S1 Fig Determination of the half-maximal inhibitory concentration (IC₅₀) of F1, F3, and the F1/F3 combination in U87 and U118 glioblastoma cells. **(A)** Cell viability of U87 cells treated with increasing concentrations of F1 for 24 h, measured by MTT assay. **(B)** Cell viability of U87 cells treated with increasing concentrations of F3 for 24 h. **(C)** Cell viability of U87 cells treated with increasing concentrations of the F1/F3 combination for 24 h. **(D)** Cell viability of U118 cells treated with increasing concentrations of the F1/F3 combination for 24 h. IC₅₀ values were calculated by nonlinear regression analysis and are indicated in each panel. Data are presented as mean ± SEM from at least three independent experiments. Statistical significance was determined using one-way ANOVA with appropriate multiple-comparison testing. *P < 0.05, **P < 0.01, ***P < 0.001, ****P < 0.0001.
(TIF)

S2 Fig Cytokine secretion in U87 and U118 cells after F1/F3 treatment. IL-18 secretion in **(A)** U87 and **(B)** U118 cells after 24 h treatment with F1/F3, measured by ELISA. TNF-α secretion in **(C)** U87 and **(D)** U118 cells after 24 h treatment with F1/F3, measured by ELISA. Data are presented as mean ± SEM from at least three independent experiments. Neither cytokine was detectably induced following F1/F3 treatment.
(TIF)

S3 Fig F1/F3 modulates splenic immune cell populations in NSG mice. **(A)** Frequency of macrophages (F4/80 ⁺ cells) among CD11b⁺ splenic myeloid cells, determined by flow cytometry. **(B)** Frequency of Ly6C⁺ cells among splenic F4/80 ⁺ macrophages. **(C)** Frequency of neutrophils (Ly6G⁺ cells) among CD11b⁺ splenic myeloid cells. Data are presented as mean ± SD from mice treated with PBS, P3, or F1/F3. Statistical significance was determined using one-way ANOVA with appropriate multiple-comparison testing. ns, not significant; *P < 0.05, **P < 0.01, ***P < 0.001, ****P < 0.0001.
(TIF)

S4 Fig Human immune cell reconstitution in PBMC-humanized NSG mice. (A) Flow cytometry detection of human CD45^+^ cells in mouse spleens after PBMC injection.(B) Proportion of human CD3e^+^ cells among CD45^+^ cells in the spleen.
(TIF)

S1 File The raw blot/gel image data. (DOCX)
