# Supplementary material for: Caerin 1.1 and 1.9 inhibit glioblastoma growth associated with modulation of the ARHGAP26-β-catenin axis and enhancing intratumoral CD8+ T cell infiltration
Source: PLoS One. 2026 Jul 9;21(7):e0353182. doi: 10.1371/journal.pone.0353182 (PMC13349103; doi:10.1371/journal.pone.0353182)
Supplement: S3 Table — (DOCX) [file pone.0353182.s003.docx]

**S1** **Table.** Human apoptosis-, pyroptosis-, PI3K-AKT-, and MAPK signaling-related genes included in the qPCR arrays

| Pyroptosis Signaling Related Genes | Apoptosis Signaling Related Genes | PI3K-AKT Signaling Related Genes | MAPK Signaling Related Genes |
| --- | --- | --- | --- |
| GSDMA,GSDMC,GSDMD,GSDME,IL18,IL1b,IL6,CASP1,CASP3,CASP4,CASP7,CASP9,CASP14,  AIM2,NLRP1,NLRP3,NLRP9 | BAD,BAK1,BAX,BCL10,BCL2L11,BID,BIK,BNIP3,BNIP3L,CD27,CD70,CYCS,DFFA,FASLG,GADD45A,HRK,LTA,NOD1,PYCARD,TNFRSF9,TNFSF10,TNFSF8,TP53BP2,TRAF3,AKT1,BAG1,BAG3,BCL2,BCL2A1,BCL2L1,BCL2L10,BCL2L2,BFAR,BIRC3,BIRC5,BIRC6,BNIP2,BRAF,CD40LG,CFLAR,IGF1R,IL10,MCL1,NAIP,NFKB1,NOL3,RIPK2,XIAP,CRADD,DAPK1,FADD,TNFRSF10A,TNFRSF10B,TNFRSF11B,TNFRSF1A,TNFRSF1B,TNFRSF21,TNFRSF25,TRADD,TNF,FAS,CASP1,CASP2,CASP3,CASP4,CASP5,CASP6,CASP7,CASP8,CASP9,CASP10,CASP14. | AKT1,AKT2,AKT3,BTK,CD4,CD40,GRB10,GRB2,HSPB1,ILK,MTCP1,PAK1,PDK2,PDPK1,PIK3CA,PIK3CG,PIK3R1,PIK3R2,PRKCA,PRKCB,PRKCZ,PTEN,TCL1A,CSNK2A1,ELK1,FOS,GRB2,HRAS,IGF1,IGF1R,IGF2,IGF2R,IRS1,JUN,MAP2K1,MAPK3,MAPK8,PTPN11,RAF1,RASA1,SHC1,SOS1,SRF,AKT1,APC,CCND1,CD14,CTNNB1,EIF2AK2,GJA1,GSK3B,IRAK1,MYD88,NFKB1,PDK1,TIRAP,TLR4,TOLLIP,CDC42,PDGFRA,RAC1,RHOA,WASL,AKT1,CDKN1B,FASLG,FOXO3,GRB2,ILK,ITGB1,MAPK1,MAPK3,PDK1,PDK2,PTEN,PTK2,RBL2,SHC1,SOS1,AKT1,BAD,GRB2,HRAS,IGF1R,IRS1,MAP2K1,MAPK1,MAPK3,RAF1,RPS6KA1,SHC1,SOS1,WHAH,AKT1,EIF4E,EIF4EBP1,EIF4G1,IRS1,MAPK1,MAPK14,MAPK3,MTOR,PABPC1,PDK1,PDK2,PRKCA,PTEN,AKT1,EIF4B,EIF4E,EIF4EBP1 ,EIF4G1,FKBP1A,MTOR,PDK1,PDK2,PTEN,RHEB,RPS6KB1,TSC1,TSC2,RPS6KB1,CASP9,CHUK,FOXO1,NFKBIA. | ARAF,BRAF,DLK1,MAP2K1,MAP2K2,MAP2K3,MAP2K4,MAP2K5,MAP2K6,MAP2K7,MAP3K1,MAP3K2,MAP3K3,MAP3K4,MAP3K5,MAP4K1,MAPK1,MAPK10,MAPK11,MAPK12,MAPK13,MAPK14,MAPK3,MAPK6,MAPK7,MAPK8,MAPK9,MOS,MST1,PAK1,PAK2,PAK3,RAF1,ATF2,CREB1,CREBBP,EGFR,ELK1,ETS1,ETS2,JUN,MAPKAPK2,MAPKAPK3,MAX,MEF2C,MKNK1,MYC,NFATC4,PRDX6,SMAD4,TP53,TBP,COL1A1,EGR1,FOS,HSPA5,HSPB1,JUN,MYC,TP53,HRAS,KRAS,KSR1,MAP2K1,MAP2K2,NRAS,CDC42,CHUK,GRB2,HRAS,KRAS,MAP2K1,MAP2K2,MAP2K4,MAP2K7,MAP4K1,MST1,NRAS,RAC1,SFN,LAMTOR3,MAP3K1,MAPK8IP2,CCNA1,CCNA2,CCNB1,CCNB2,CCND1,CCND2,CCND3,CCNE1,CDK2,CDK4,CDK6,CDKN1A,CDKN1B,CDKN1C,CDKN2A,CDKN2B,CDKN2C,CDKN2D,E2F1,RB1,DUSP2,DUSP4. |
