# Supplementary material for: Caerin 1.1 and 1.9 inhibit glioblastoma growth associated with modulation of the ARHGAP26-β-catenin axis and enhancing intratumoral CD8+ T cell infiltration
Source: PLoS One. 2026 Jul 9;21(7):e0353182. doi: 10.1371/journal.pone.0353182 (PMC13349103; doi:10.1371/journal.pone.0353182)
Supplement: S4 Table — (DOCX) [file pone.0353182.s004.docx]

**S2 Table.** Antibodies used for flow cytometry analysis**.**

| **Antibody** | **Label** | **Article Number** | **Clone** | **Provider** |
| --- | --- | --- | --- | --- |
| CD45.2 | FITC | 11-0454-85 | 104 | eBioscience |
| F4/80 | PE | 12-4801-82 | BM8 | eBioscience |
| CD11b | Percp-cy5.5 | 45-0112-82 | M1/70 | eBioscience |
| Ly6G | APC | 17-9668-82 | 1A8-Ly6g | eBioscience |
| Ly6C | BV421 | 562727 | AL-21 | BD Biosciences |
| CD3e | APC-cy7 | 557596 | 145-2C11 | BD Biosciences |
| CD8a | PE-cy7 | 552877 | 53-6.7 | BD Biosciences |
| CD4 | Percp-cy5.5 | 45-0042-82 | RM4-5 | eBioscience |
| Human PD-L1（CD274） | PE-cy7 | 25-5983-42 | 29E.2A3 | eBioscience |
| Human CD45 | FITC | 304006 | 2D1 | Biolegend |
| Human CD3 | PE | 300308 | HIT3a | Biolegend |
| Human CD8a | Percp-cy5.5 | 301028 | RPA-T8 | Biolegend |
| Human CD4 | APC-cy7 | 300514 | SK3 | Biolegend |
| Human CD68 | PE | 333806 | Y1/82A | Biolegend |
| Fixable Viability Stain 510 | - | 564406 | - | BD Biosciences |
