# Supplementary figures and images for: Caerin 1.1 and 1.9 inhibit glioblastoma growth associated with modulation of the ARHGAP26-β-catenin axis and enhancing intratumoral CD8+ T cell infiltration
Source: PLoS One. 2026 Jul 9;21(7):e0353182. doi: 10.1371/journal.pone.0353182 (PMC13349103; doi:10.1371/journal.pone.0353182)

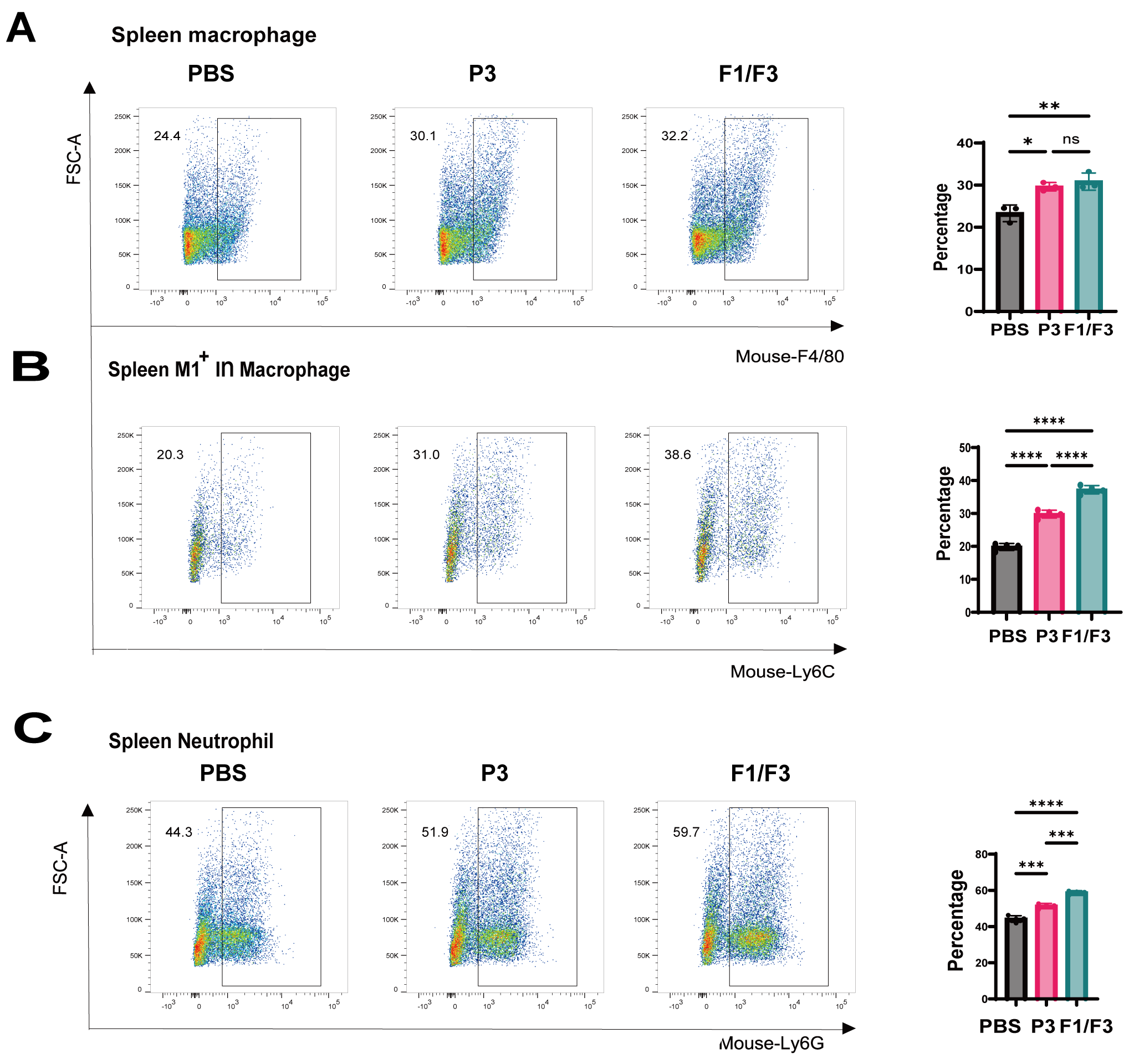

Supplement: S3 Fig — (A) Frequency of macrophages (F4/80 ⁺ cells) among CD11b⁺ splenic myeloid cells, determined by flow cytometry. (B) Frequency of Ly6C⁺ cells among splenic F4/80 ⁺ macrophages. (C) Frequency of neutrophils (Ly6G⁺ cells) among CD11b⁺ splenic myeloid cells. Data are presented as mean ± SD from mice treated with PBS, P3, or F1/F3. Statistical significance was determined using one-way ANOVA with appropriate multiple-comparison testing. ns, not significant; *P < 0.05, **P < 0.01, ***P < 0.001, ****P < 0.0001. (TIF) [file pone.0353182.s007.tif]

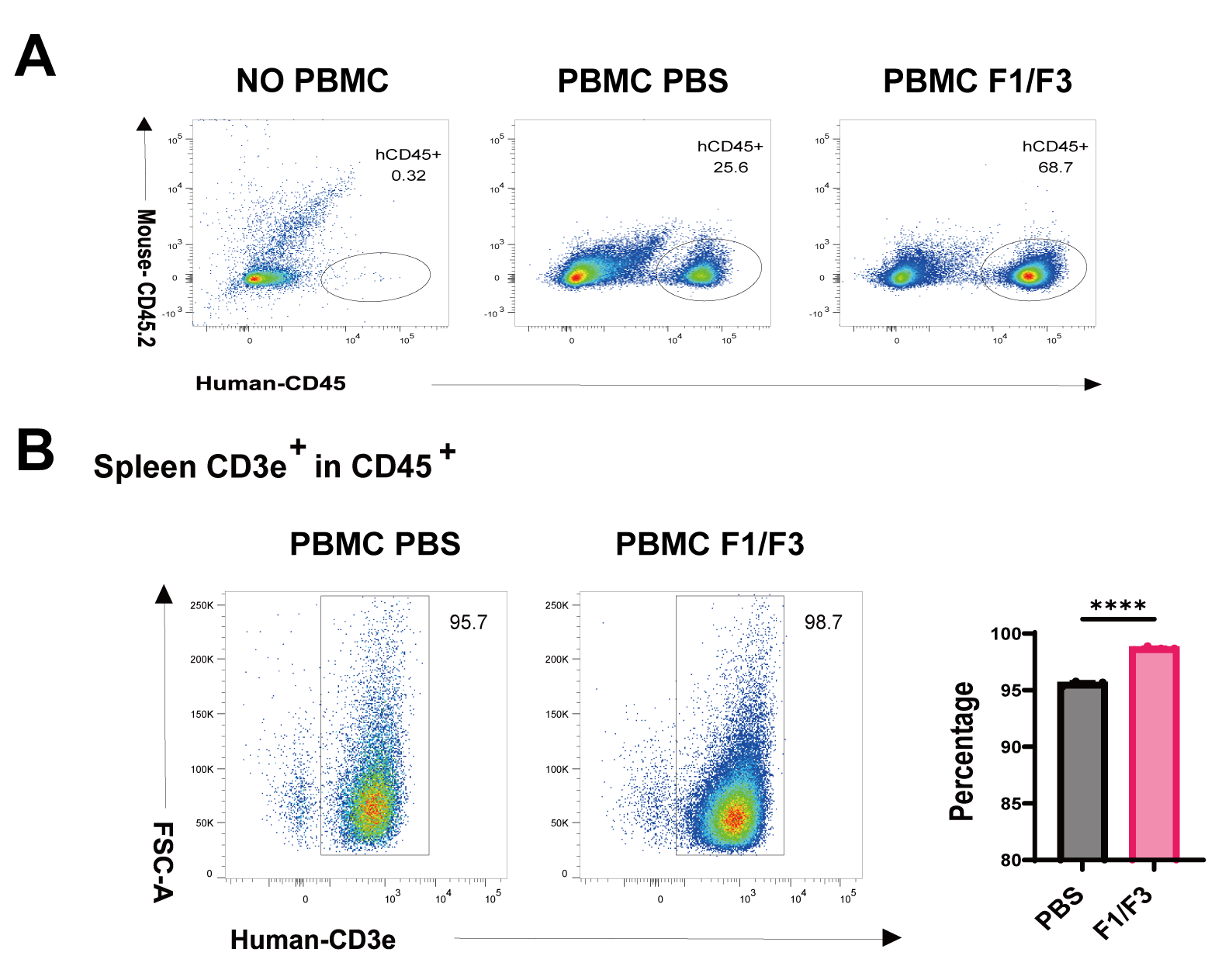

Supplement: S4 Fig — (A) Flow cytometry detection of human CD45+ cells in mouse spleens after PBMC injection.(B) Proportion of human CD3e+ cells among CD45+ cells in the spleen. (TIF) [file pone.0353182.s008.tif]

**WB original images**

ARHGAP26


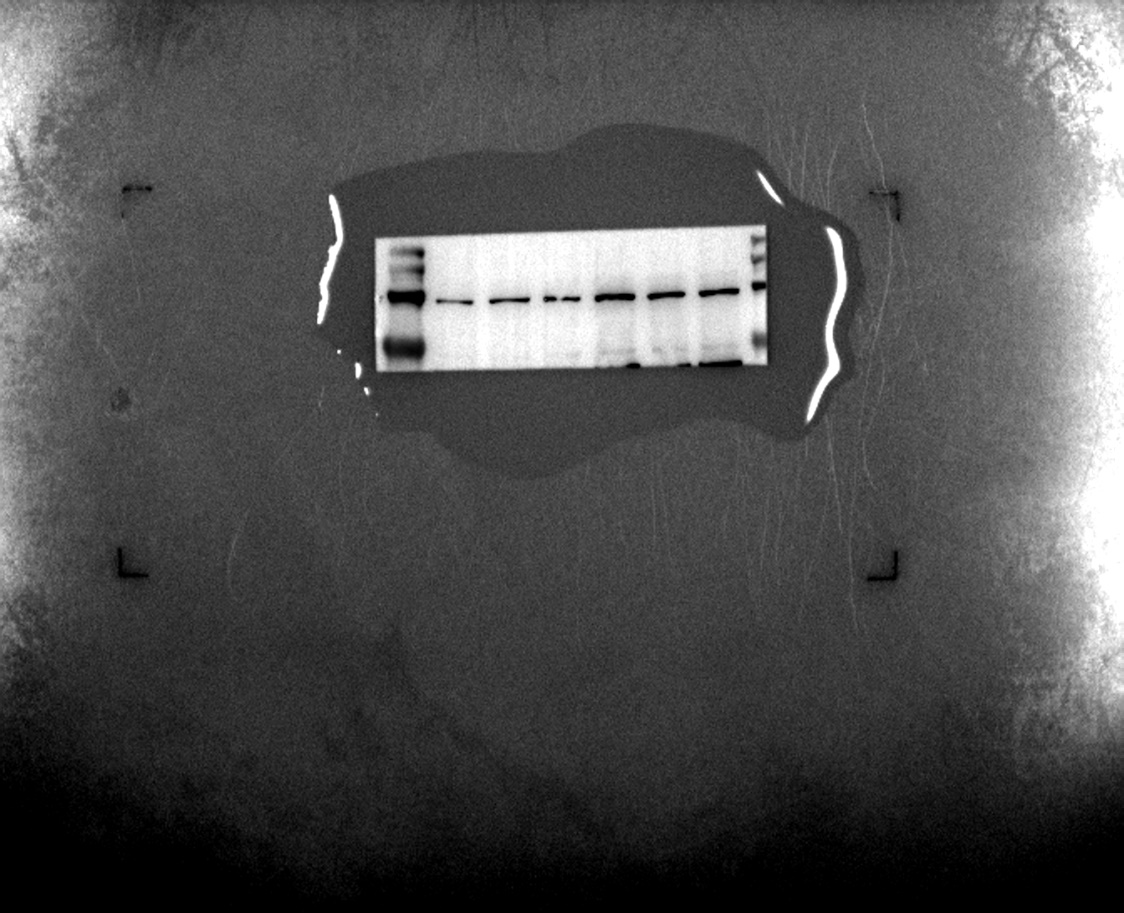




GAPDH


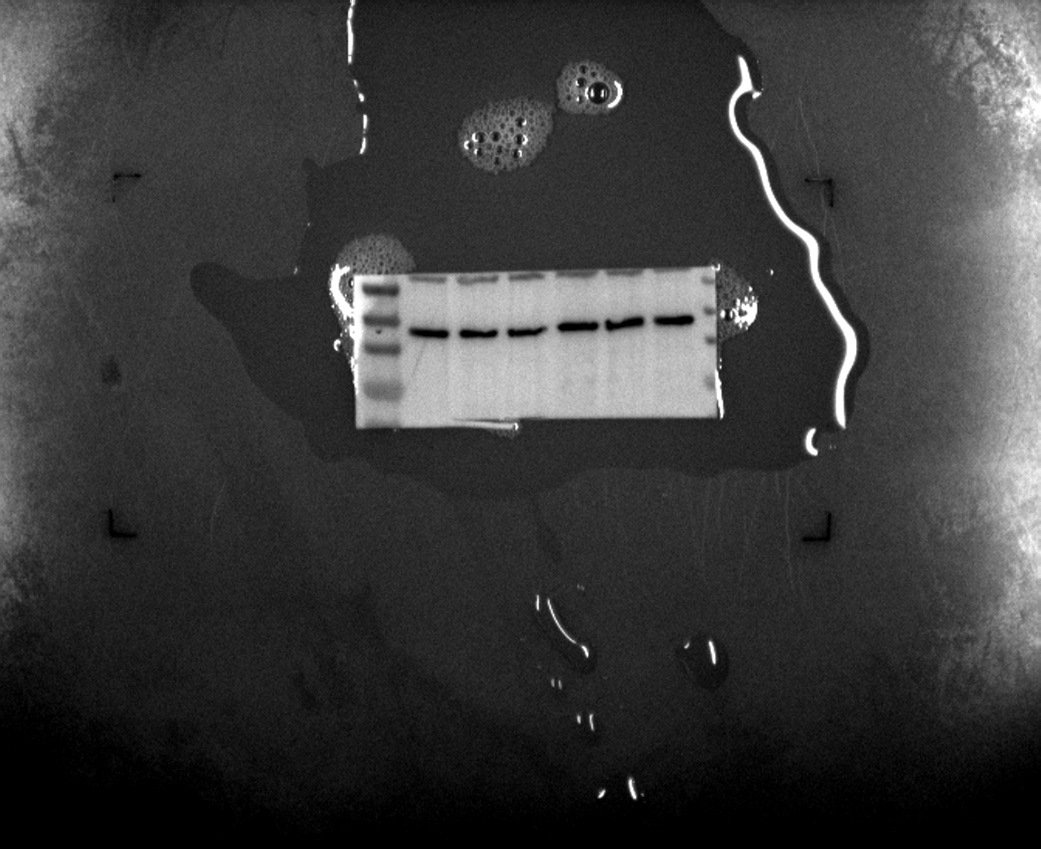

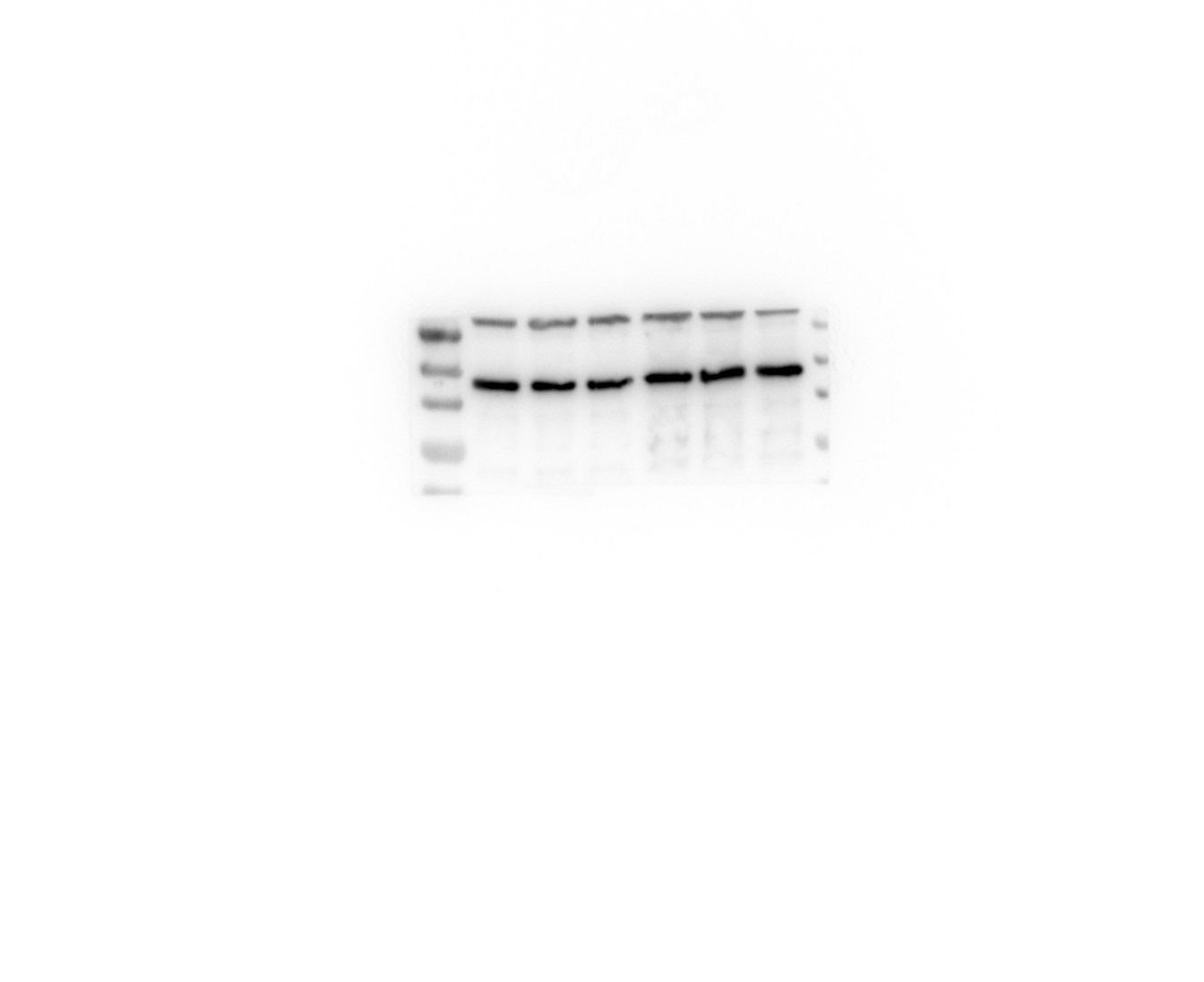


MMP2


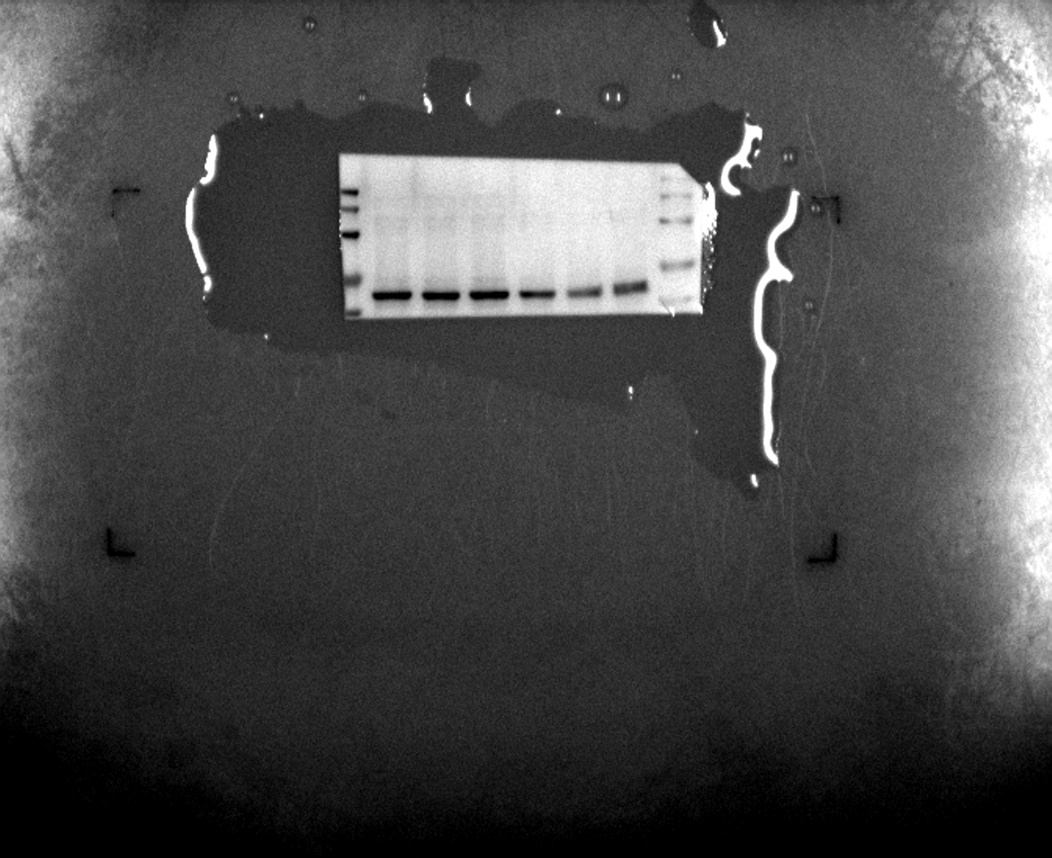




GAPDH


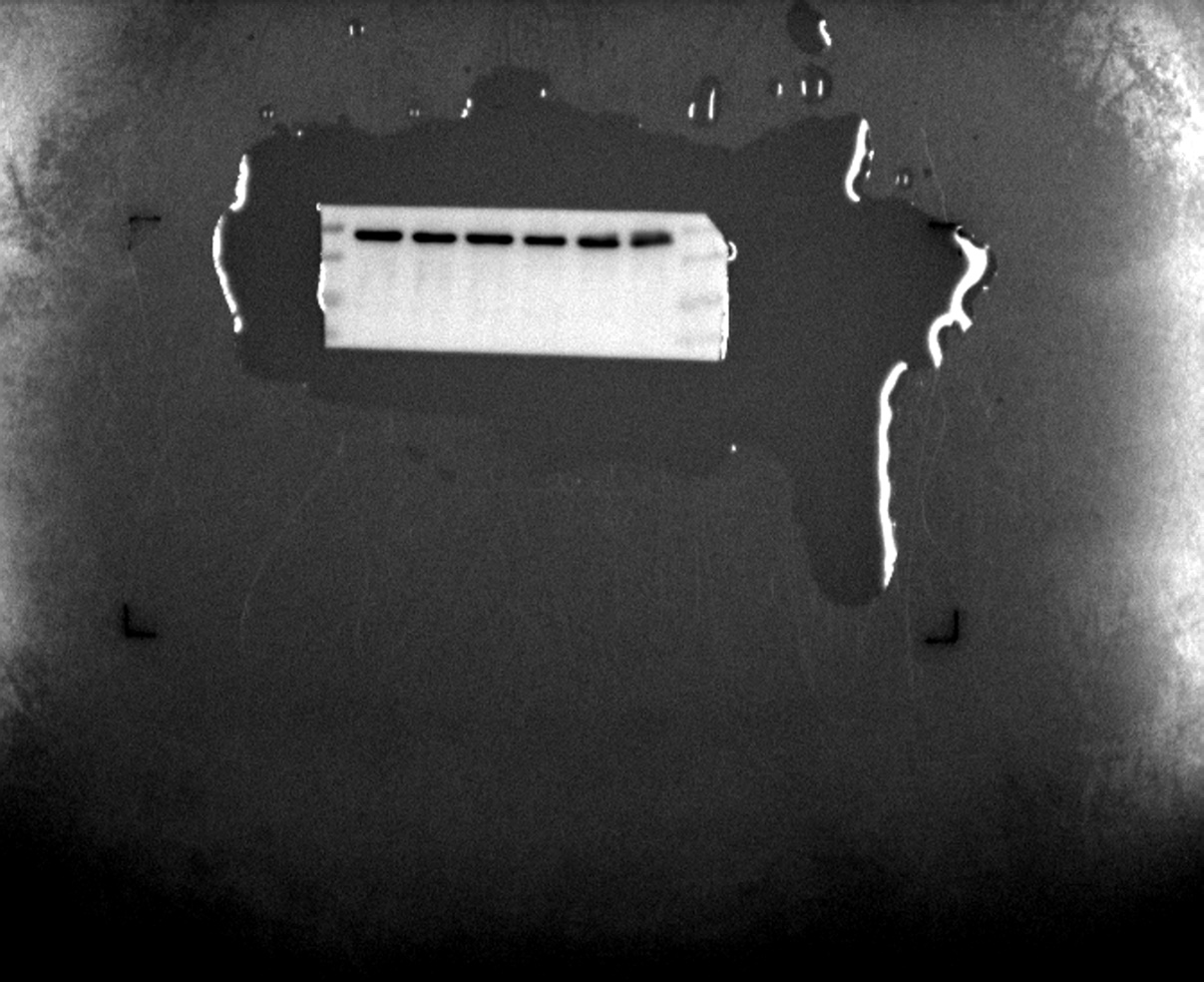

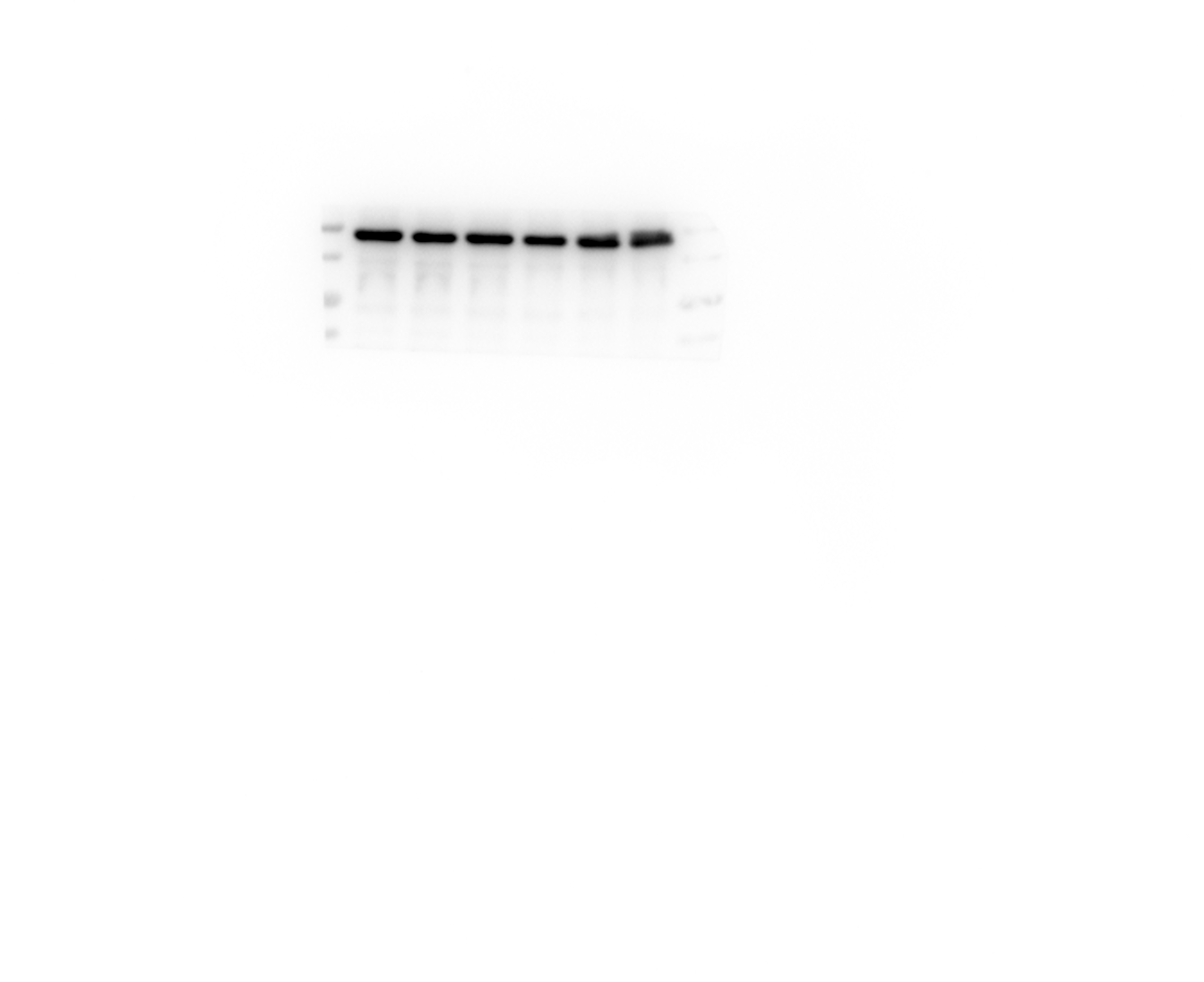


MMP7


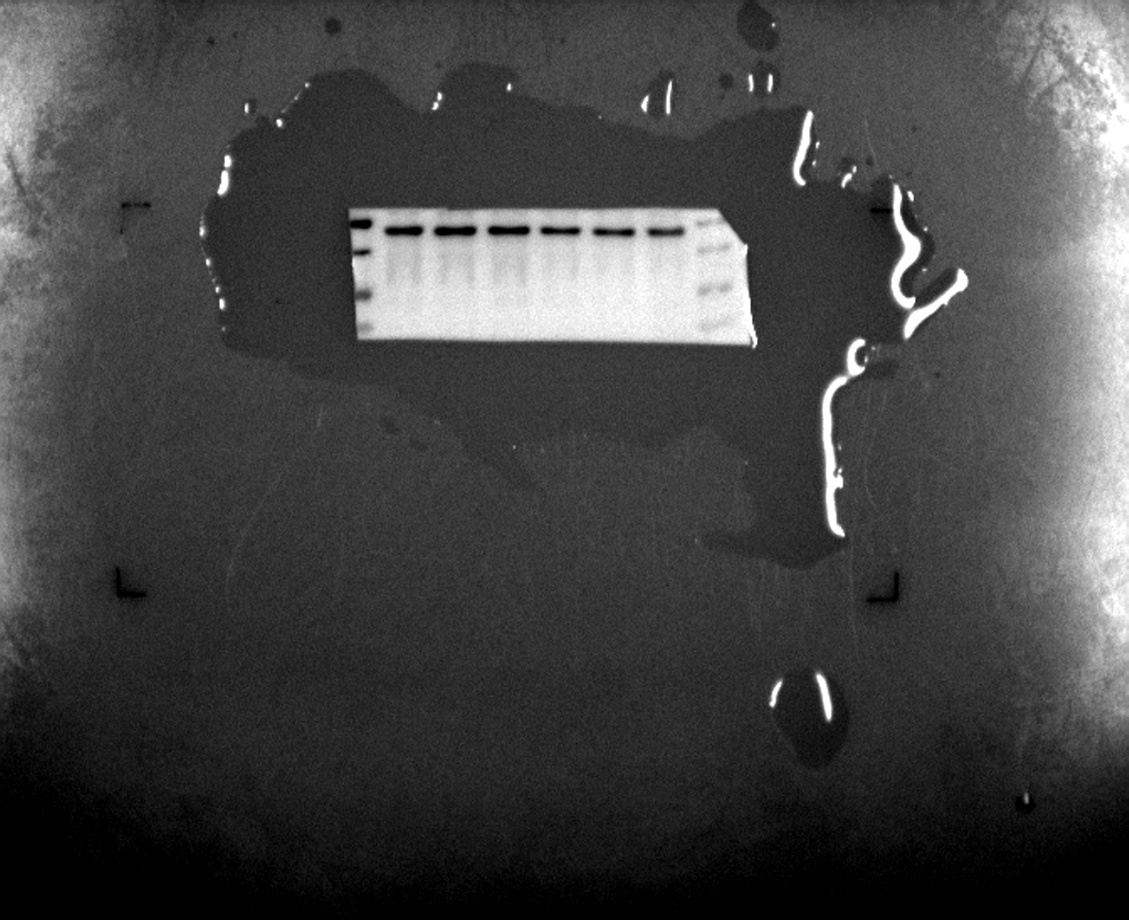

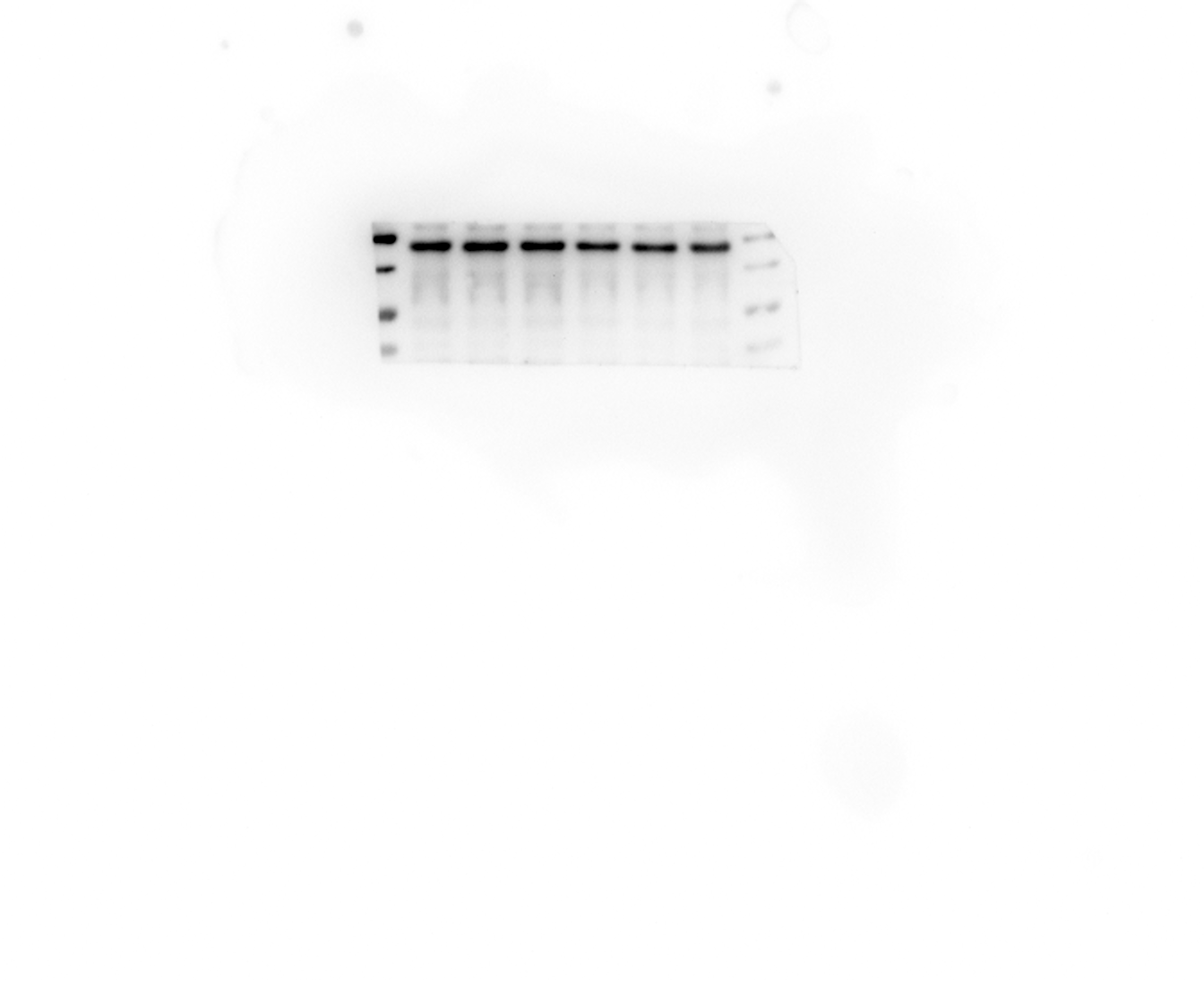


Tublin


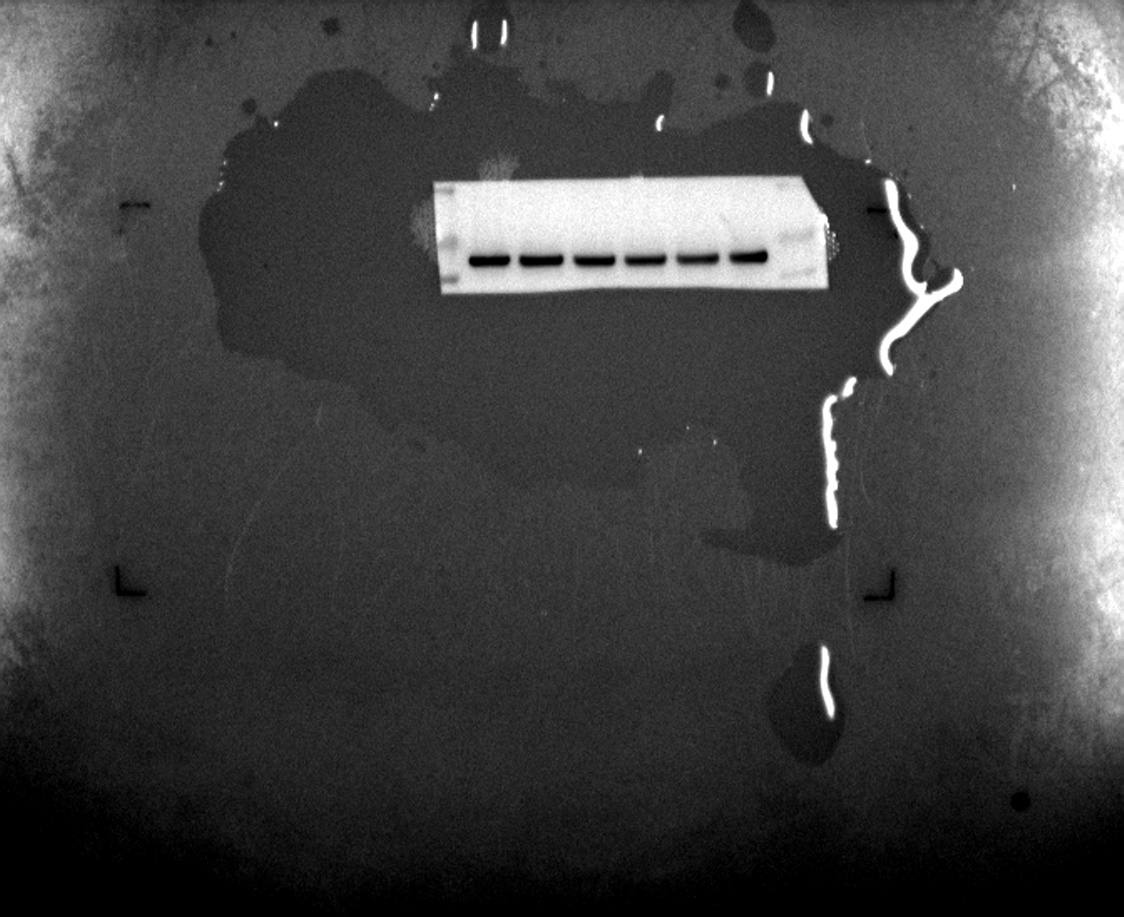




VEGFA


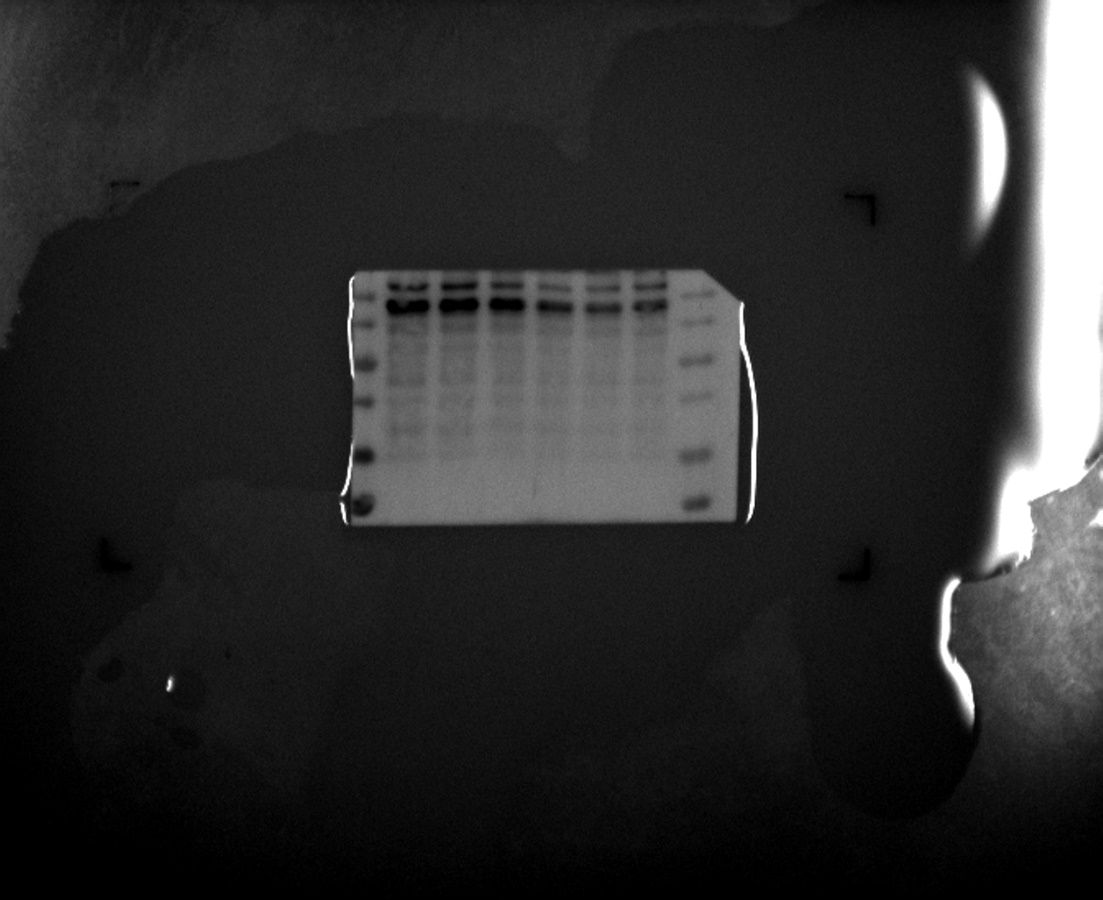




Tublin


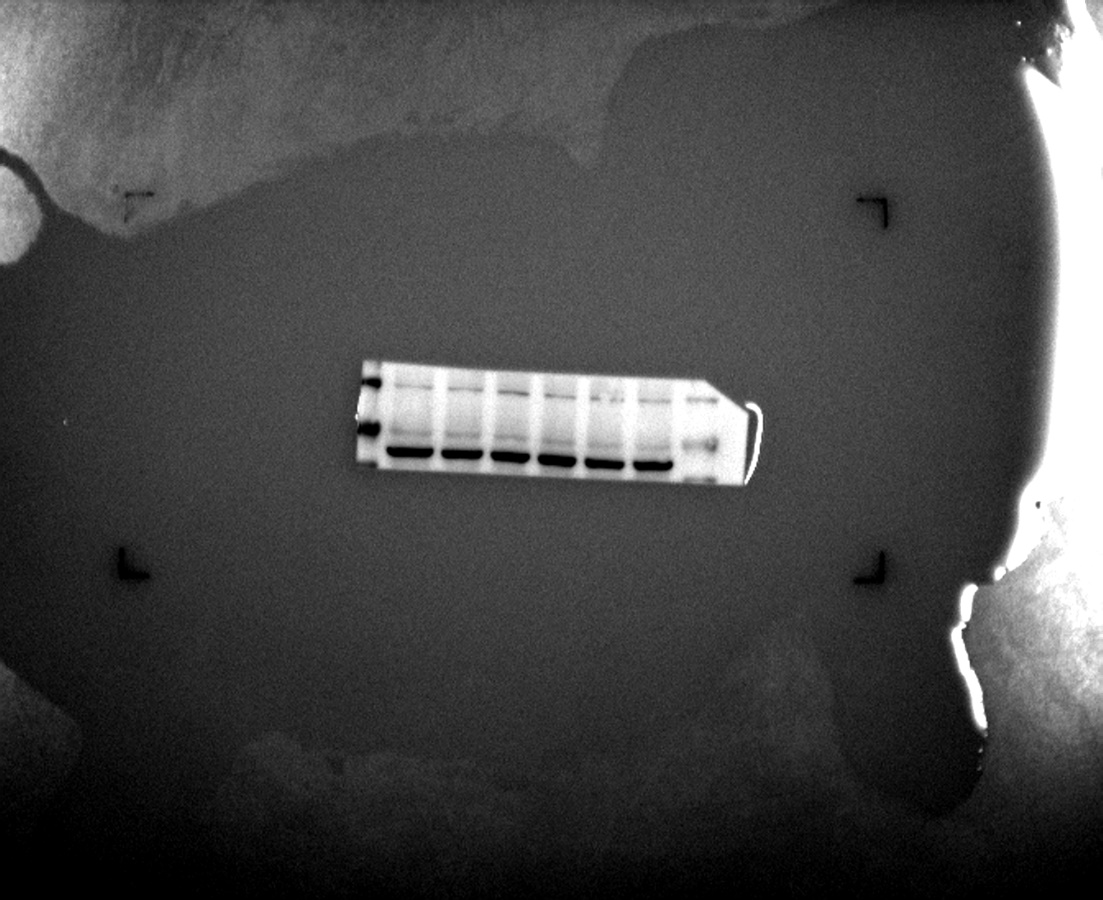




β-catenin


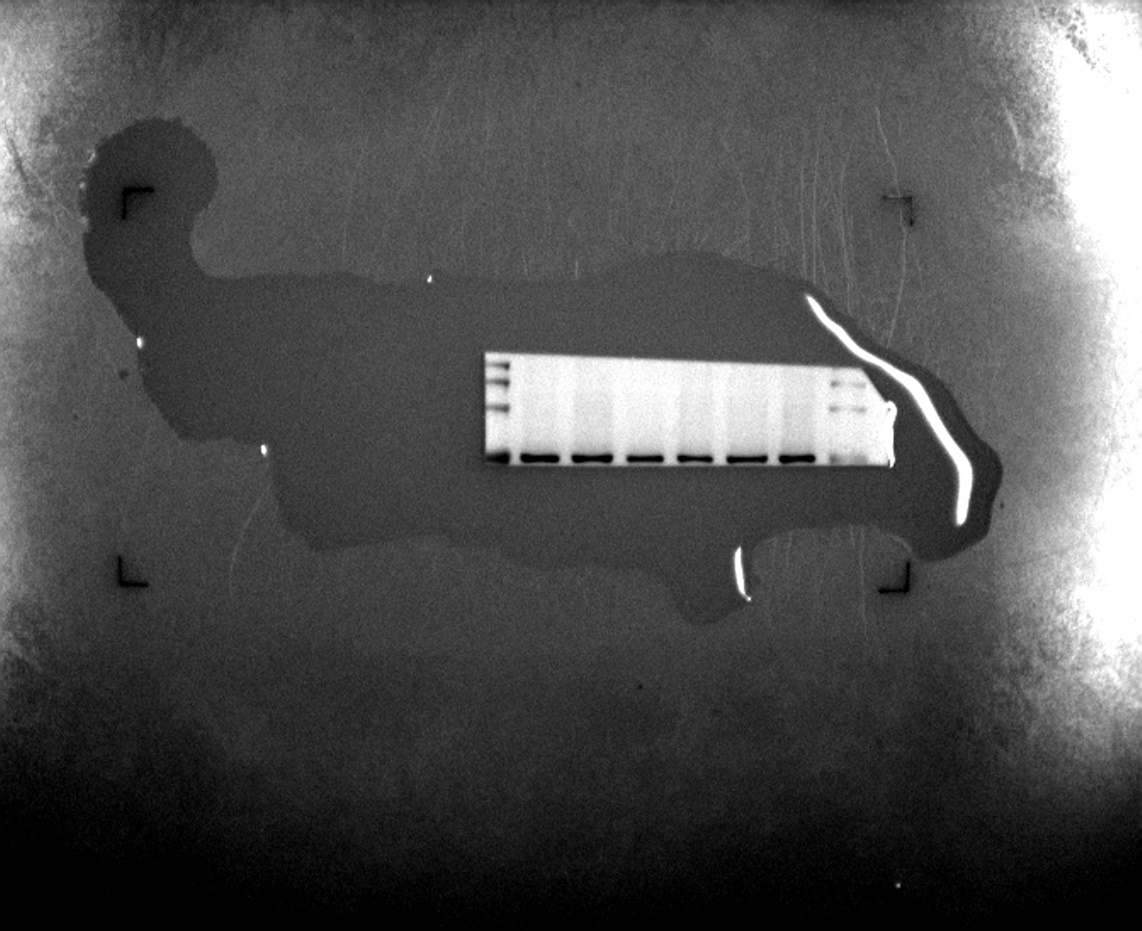




Tublin


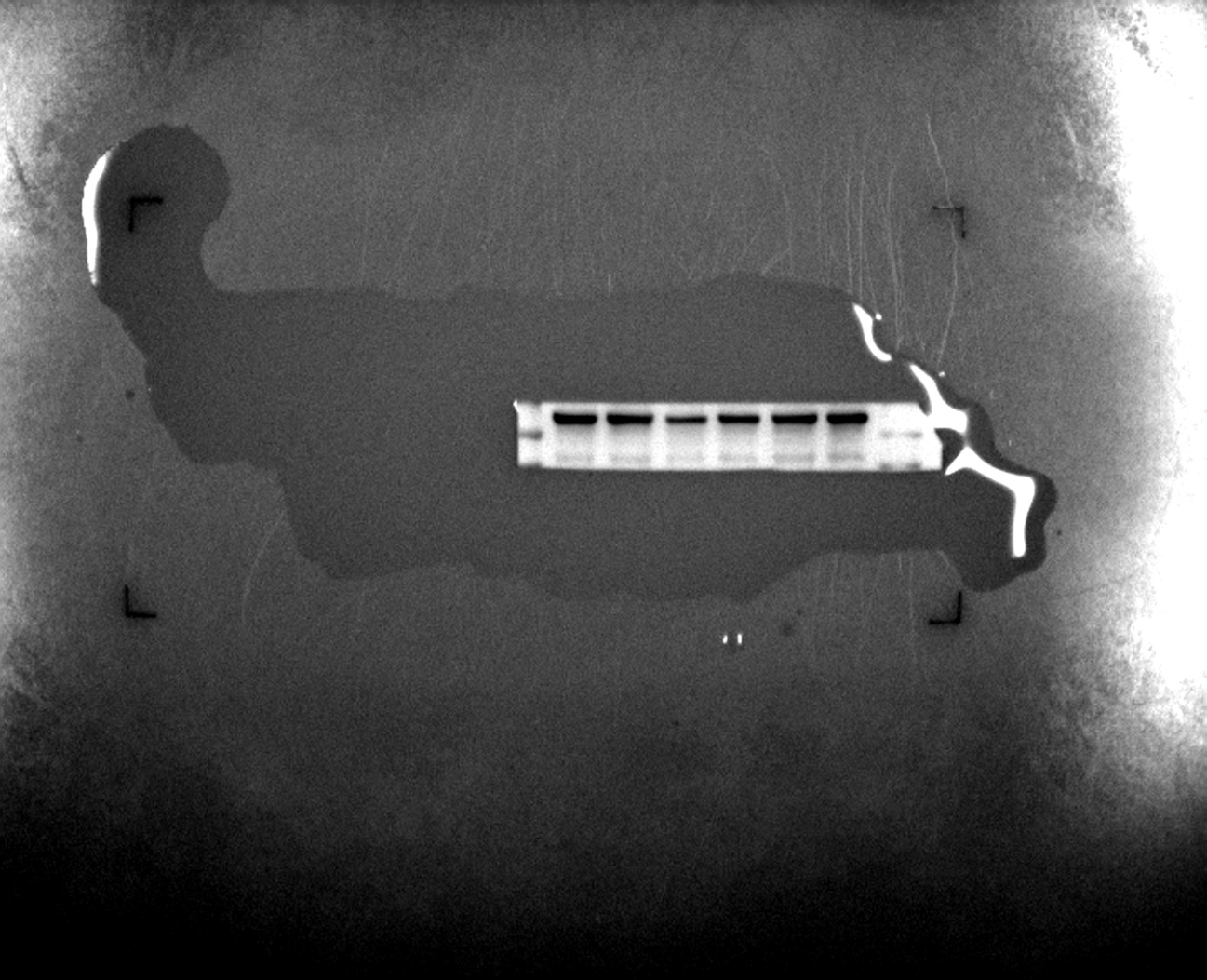

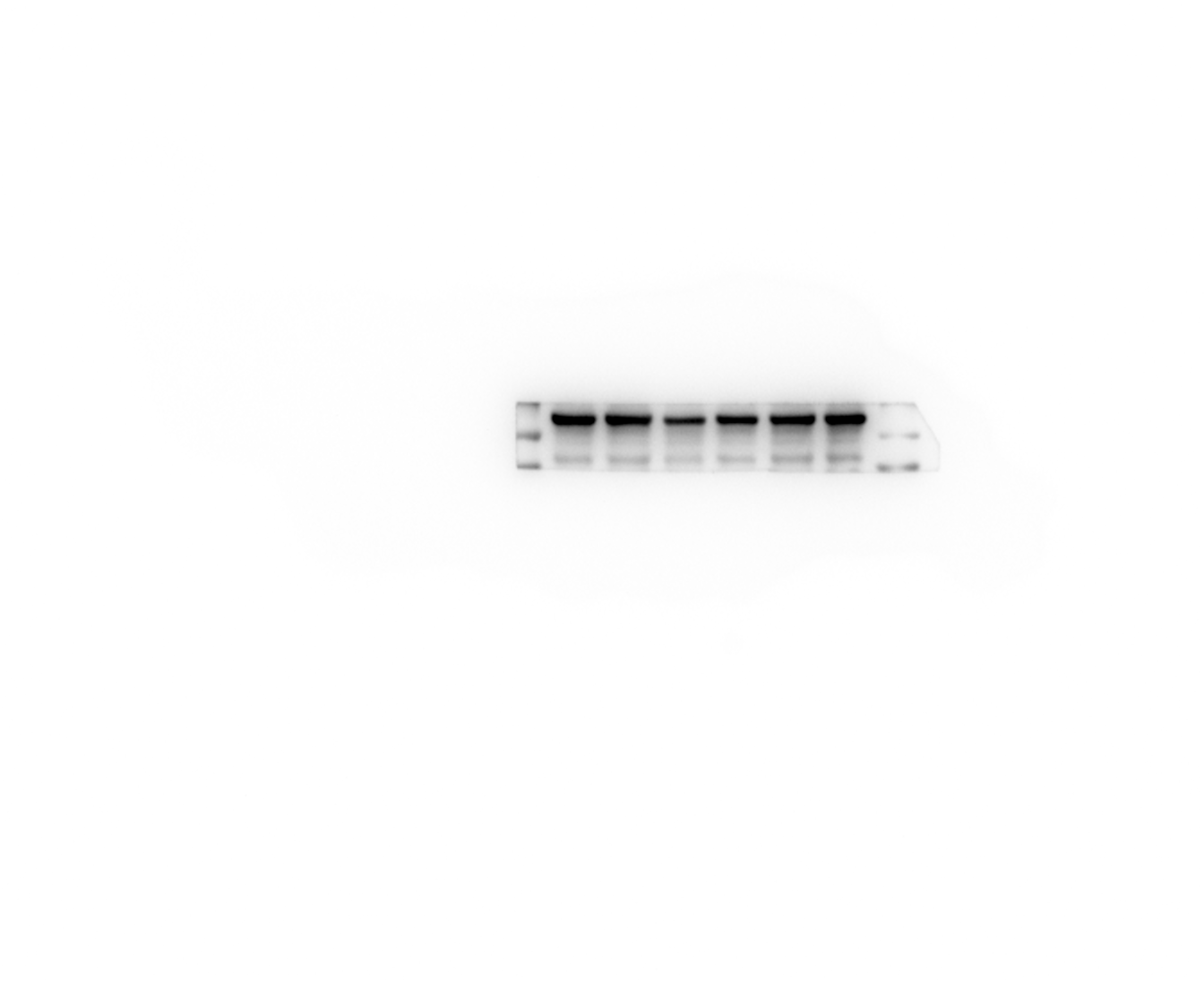

Supplement: S1 File — (DOCX) [file pone.0353182.s009.docx]
