# Supplementary material for: Caerin 1.1 and 1.9 inhibit glioblastoma growth associated with modulation of the ARHGAP26-β-catenin axis and enhancing intratumoral CD8+ T cell infiltration
Source: PLoS One. 2026 Jul 9;21(7):e0353182. doi: 10.1371/journal.pone.0353182 (PMC13349103; doi:10.1371/journal.pone.0353182)
Supplement: Attachment — Submitted filename: Comments_PlosONE.docx [file pone.0353182.s010.docx]

This manuscript titled, “Caerin 1.1 and 1.9 inhibit glioblastoma growth by regulating the ARHGAP26–β-catenin axis and enhancing intratumoral CD8⁺ T cell infiltration” investigates the anti-tumor effects of caerin peptides (1.1 and 1.9) in glioblastoma using in vitro assays, xenograft models, and a PBMC-humanized mouse model. The study addresses an important clinical challenge in glioblastoma and presents a multi-layered experimental approach. The integration of tumor-intrinsic signaling and immune modulation is a strength. However, several issues regarding **mechanistic rigor, data interpretation, redundancy, and clarity** need to be considered before the manuscript can be considered for publication.

Major Comments:

1. The central claim that ARHGAP26 mediates the anti-tumor effects is not fully supported. The study shows correlation, however, no causal experiments are conducted. There is no knockdown or overexpression validation of ARHGAP26.
2. The manuscript concludes that caerin peptides induce “atypical pyroptosis.” However, IL-1β and IL-18 secretion is minimal or absent, Key pyroptosis effectors (e.g., GSDMD) are downregulated and no direct evidence of inflammasome activation is provided. Hence, The data do not convincingly support pyroptosis as a major mechanism.
3. The manuscript states that apoptosis is the primary mechanism of cell death based on gene expression data. But there is no functional validation like caspase inhibitor assay or annexinV/PI quantification. Authors should perform additional validation experiments to confirm the mechanism of cell death
4. The PBMC-humanized mouse experiments use very small group sizes (n=3). This limits statistical power, weakens conclusions about immune mechanisms. Authors should increase the sample size or explicitly acknowledge this limitation in the Discussion.
5. The study shows increased CD8⁺ T cell infiltration but does not assess their functional state. Increased infiltration does not necessarily imply enhanced anti-tumor activity. Authors should include functional assays to support their claim

Minor Concerns

1. The description of PCR array results is excessively long and appears twice (Results and Discussion). Summarize key findings concisely in the main text.
2. Several grammatical errors and awkward phrases are present.
3. The number of replicates and independent experiments is not always clearly stated. Clarify whether data represent biological or technical replicates.
4. Ensure all figures include exact n values, statistical tests used and clear labeling of error bars
5. Use consistent terminology for pyroptosis-like death and immune microenvironment remodeling
