# Supplementary material for: Caerin 1.1 and 1.9 inhibit glioblastoma growth associated with modulation of the ARHGAP26-β-catenin axis and enhancing intratumoral CD8+ T cell infiltration
Source: PLoS One. 2026 Jul 9;21(7):e0353182. doi: 10.1371/journal.pone.0353182 (PMC13349103; doi:10.1371/journal.pone.0353182)
Supplement: Attachment — Submitted filename: Response to reviewer.docx [file pone.0353182.s011.docx]

Dear Editor and Reviewers,

We sincerely thank you for your careful evaluation of our manuscript and for the constructive and insightful comments provided. We have carefully considered all suggestions and revised the manuscript accordingly to improve its clarity, scientific rigor, and overall presentation. In the revised manuscript, all modifications have been highlighted in red fonts, and detailed point-by-point responses to each comment are provided below.

**Editor**

1. Please ensure that your manuscript meets PLOS ONE's style requirements, including those for file naming.

RESPONSE: We have carefully reviewed the manuscript and revised the formatting, file naming, and presentation to ensure compliance with all PLOS ONE style requirements.

2. To comply with PLOS One submissions requirements, in your Methods section, please provide additional information regarding the experiments involving animals and ensure you have included details on (1) methods of anesthesia and/or analgesia, and (2) efforts to alleviate suffering.

RESPONSE: We have revised the Methods section to provide additional details regarding the animal experiments, including the methods of anesthesia and euthanasia, humane endpoint criteria, daily monitoring procedures, and measures taken to minimize animal suffering and distress throughout the study. These additions were incorporated to ensure compliance with PLOS ONE animal research reporting requirements and ARRIVE guidelines.

3. Please provide an amended Funding Statement declaring this commercial affiliation, as well as a statement regarding the Role of Funders in your study. If the funding organization did not play a role in the study design, data collection and analysis, decision to publish, or preparation of the manuscript and only provided financial support in the form of authors' salaries and/or research materials, please review your statements relating to the author contributions, and ensure you have specifically and accurately indicated the role(s) that these authors had in your study. You can update author roles in the Author Contributions section of the online submission form. Please also include the following statement within your amended Funding Statement.

RESPONSE: We have amended the Funding Statement to clarify the commercial affiliation and the role of the funder in this study. Specifically, we added the following statement: “The funder provided support in the form of salaries for authors [JL, YL, QF, XL, GN], but did not have any additional role in the study design, data collection and analysis, decision to publish, or preparation of the manuscript. The specific roles of these authors are articulated in the ‘Author Contributions’ section.” We have also reviewed and updated the Author Contributions section in the online submission system to ensure that the contributions of all authors are accurately described

4. Please also provide an updated Competing Interests Statement declaring this commercial affiliation along with any other relevant declarations relating to employment, consultancy, patents, products in development, or marketed products, etc. Within your Competing Interests Statement, please confirm that this commercial affiliation does not alter your adherence to all PLOS ONE policies on sharing data and materials by including the following statement: "This does not alter our adherence to PLOS ONE policies on sharing data and materials.”

RESPONSE: We have updated the Competing Interests Statement to declare the relevant commercial affiliation and related author associations. We also confirmed that these affiliations do not influence compliance with the journal’s policies on data and material sharing. Specifically, we included the statement: “This does not alter our adherence to PLOS ONE policies on sharing data and materials.” In addition, we carefully reviewed the manuscript to ensure that all relevant declarations regarding employment, affiliations, and potential competing interests were appropriately disclosed。

5. PLOS ONE now requires that authors provide the original uncropped and unadjusted images underlying all blot or gel results reported in a submission’s figures or Supporting Information files. In your cover letter, please note whether your blot/gel image data are in Supporting Information or posted at a public data repository, provide the repository URL if relevant, and provide specific details as to which raw blot/gel images, if any, are not available.

RESPONSE: We have provided the original uncropped and unadjusted blot/gel images underlying the relevant figures as Supporting Information files in accordance with PLOS ONE requirements. We have also updated the cover letter to indicate that the raw blot/gel image data are included in the Supporting Information **S1 File**. All available raw images have been provided, and no relevant blot/gel image data have been intentionally omitted.

6. We note that your Data Availability Statement is currently as follows: All relevant data are within the manuscript and in Supporting Information files. Please confirm at this time whether or not your submission contains all raw data required to replicate the results of your study.

RESPONSE: We confirm that the submission contains all raw data required to replicate the findings of this study. All relevant experimental data, including raw blot/gel images and supporting datasets, have been provided within the manuscript and Supporting Information files in accordance with PLOS ONE data availability requirements.

**Reviewer #1**: 
1. The central claim that ARHGAP26 mediates the anti-tumor effects is not fully supported. The study shows correlation, however, no causal experiments are conducted. There is no knockdown or overexpression validation of ARHGAP26.

RESPONSE: We thank the reviewer for this important comment. We agree that the current study primarily demonstrates an association between F1/F3 treatment and modulation of the ARHGAP26–β-catenin axis, rather than definitive causal evidence. In the present work, ARHGAP26 was identified through our proteomic profiling and subsequently validated by western blotting together with downstream β-catenin-related proteins, including MMP2, MMP7, and VEGFA. These findings suggest that ARHGAP26 is likely involved in the antitumor effects of F1/F3 in glioblastoma cells.

However, we acknowledge that direct mechanistic validation using ARHGAP26 knockdown or overexpression experiments would further strengthen the causal interpretation. Due to time limitations and the extensive additional experiments required, these studies were beyond the scope of the current manuscript. We have therefore revised the manuscript to moderate the mechanistic claims and clarify that our data support a strong association between F1/F3 treatment and regulation of the ARHGAP26–β-catenin signaling pathway, rather than definitive proof of causality. We have also added this limitation in the Discussion section and highlighted ARHGAP26 functional validation as an important direction for future studies.

In addition, to avoid overstating mechanistic causality, we revised the manuscript title and related text throughout the manuscript to reflect that the observed changes in the ARHGAP26–β-catenin axis are associated with, rather than definitively proven to mediate, the antitumor effects of F1/F3.

2. The manuscript concludes that caerin peptides induce “atypical pyroptosis.” However, IL-1β and IL-18 secretion is minimal or absent, Key pyroptosis effectors (e.g., GSDMD) are downregulated and no direct evidence of inflammasome activation is provided. Hence, the data do not convincingly support pyroptosis as a major mechanism.

RESPONSE: We agree that the current data do not provide sufficient evidence to support canonical inflammasome-mediated pyroptosis as a major mechanism of F1/F3-induced cell death in glioblastoma cells. Our original interpretation was based on the substantial LDH release and membrane damage observed following F1/F3 treatment, together with the differential expression of several pyroptosis-related genes identified by PCR array analysis. However, as the reviewer correctly noted, IL-1β and IL-18 secretion was minimal or undetectable, GSDMD expression was downregulated, and no direct evidence of inflammasome activation or gasdermin cleavage was provided.

We therefore agree that the evidence does not support the conclusion that pyroptosis is a dominant mechanism underlying F1/F3-mediated cytotoxicity in U87 and U118 cells. In the revised manuscript, we have moderated our interpretation throughout the Results, Discussion, figure legends, and section headings. Specifically, we replaced the term “atypical pyroptosis” with more cautious wording such as “pyroptosis-associated features” or “pyroptosis-related signaling changes” where appropriate. We also revised the Results section to emphasize that apoptosis-related pathways exhibited the most extensive transcriptional changes and likely represent the predominant mechanism of cell death induced by F1/F3.

Furthermore, we have added a statement in the Discussion acknowledging that the absence of robust IL-1β/IL-18 secretion, the downregulation of canonical pyroptosis effectors, and the lack of direct inflammasome activation assays limit our ability to conclude that pyroptosis contributes substantially to the observed antitumor effects. Future studies examining gasdermin cleavage, inflammasome activation, and caspase-dependent signaling will be required to clarify the contribution of pyroptosis-related pathways to F1/F3-induced glioblastoma cell death.

Accordingly, the manuscript now presents apoptosis as the principal mechanism associated with F1/F3-induced cytotoxicity, while pyroptosis-related signaling changes are discussed as secondary observations that warrant further investigation.

3. The manuscript states that apoptosis is the primary mechanism of cell death based on gene expression data. But there is no functional validation like caspase inhibitor assay or annexinV/PI quantification. Authors should perform additional validation experiments to confirm the mechanism of cell death.

RESPONSE: We thank the reviewer for this valuable comment and agree that direct functional validation would strengthen the conclusion that apoptosis is the predominant mechanism of F1/F3-induced cell death in glioblastoma cells. In the current study, our conclusion was based on several lines of evidence, including the extensive transcriptional changes observed in apoptosis-related genes, the upregulation of both intrinsic and extrinsic apoptosis-associated pathways, and the lack of evidence supporting ferroptosis or necroptosis based on inhibitor studies. In addition, our previous studies demonstrated that F1/F3 induces caspase-3-dependent apoptosis in multiple cancer cell types, including HeLa cells and melanoma cells, providing supporting evidence for a similar mechanism in glioblastoma cells.

However, we agree that the present study does not include direct functional assays such as Annexin V/PI staining, caspase inhibition experiments, or measurement of caspase activation in U87 and U118 cells. Therefore, we have revised the manuscript to moderate our interpretation and avoid presenting apoptosis as definitively proven. Specifically, we now state that the data are consistent with apoptosis being the predominant cell death pathway rather than conclusively demonstrating it. We have also revised the Discussion section to acknowledge the lack of direct functional validation as a limitation of the current study.

We agree that additional experiments, including Annexin V/PI analysis, caspase activation assays, and caspase inhibition studies, would provide stronger mechanistic evidence and will be pursued in future investigations to further define the cell death pathways induced by F1/F3 in glioblastoma cells. "Our interpretation is also supported by our recent study demonstrating that F1/F3 induces caspase-3 activation and apoptosis-associated cell death in epithelial cancer cells (Luo et al., Sci Rep, 2025)."

4. The PBMC-humanized mouse experiments use very small group sizes (n = 3). This limits statistical power, weakens conclusions about immune mechanisms. Authors should increase the sample size or explicitly acknowledge this limitation in the Discussion.

RESPONSE: We agree that the small sample size in the PBMC-humanized mouse experiment (n = 3 per group) limits the statistical power of the study and reduces the strength of conclusions regarding the underlying immune mechanisms. The humanized NSG mouse model is technically demanding, time-consuming, and costly, particularly when establishing human immune cell engraftment and maintaining animals over an extended experimental period. Consequently, the current experiment was designed as a proof-of-concept study to evaluate whether F1/F3 could exert antitumor effects in the presence of a human immune system.

Despite the limited sample size, we observed consistent effects across multiple independent endpoints, including reduced tumor growth, increased infiltration of human CD8⁺ T cells and macrophages, and reduced PD-L1 expression within the tumor microenvironment. Nevertheless, we agree that these findings should be interpreted with caution.

We have therefore revised the Discussion section to explicitly acknowledge the limited sample size as a study limitation. Specifically, we note that the results provide preliminary evidence supporting the immunomodulatory activity of F1/F3 in a humanized glioblastoma model, but that larger studies with increased animal numbers and additional mechanistic analyses will be required to confirm these observations and more rigorously define the immune pathways involved.

In the revised manuscript, we have also moderated the language describing the immune mechanisms to avoid overinterpretation of findings derived from this exploratory study.

5. The study shows increased CD8⁺ T cell infiltration but does not assess their functional state. Increased infiltration does not necessarily imply enhanced anti-tumor activity. Authors should include functional assays to support their claim.

RESPONSE: We agree that increased CD8⁺ T-cell infiltration alone does not necessarily indicate enhanced antitumor function. Functional characterization of infiltrating CD8⁺ T cells, such as assessment of activation markers, cytokine production (e.g., IFN-γ, TNF-α), cytotoxic molecules (e.g., granzyme B, perforin), or *ex vivo* killing assays, would provide stronger evidence for enhanced antitumor activity.

In the current study, our primary objective was to determine whether F1/F3 could alter the immune landscape within the glioblastoma tumor microenvironment. We found that F1/F3 treatment was associated with increased infiltration of human CD8⁺ T cells, reduced PD-L1 expression, and significant suppression of tumor growth in the humanized NSG mouse model. While these observations are consistent with enhanced antitumor immunity, we agree that they do not directly demonstrate improved CD8⁺ T-cell functionality.

Due to limitations in sample availability and the exploratory nature of the humanized mouse experiments, functional analyses of tumor-infiltrating CD8⁺ T cells were not performed in the current study. We have therefore revised the manuscript to avoid implying that increased infiltration necessarily reflects enhanced effector function. Specifically, we now state that F1/F3 is associated with increased CD8⁺ T-cell infiltration rather than enhanced CD8⁺ T-cell activity.

In addition, we have added a statement in the Discussion acknowledging this limitation and highlighting future studies aimed at characterizing the activation, exhaustion, and cytotoxic status of tumor-infiltrating CD8⁺ T cells. Such experiments will be important for defining the mechanisms by which F1/F3 modulates antitumor immunity in glioblastoma.

6. The description of PCR array results is excessively long and appears twice (Results and Discussion). Summarize key findings concisely in the main text.

RESPONSE: We agree that the original description of the PCR array results was overly detailed and repetitive. In response, we have substantially condensed the presentation of the PCR array data in both the Results and Discussion sections. The revised Results section now focuses on the principal findings, namely that apoptosis-related genes exhibited the most extensive transcriptional changes following F1/F3 treatment, while pyroptosis-related and PI3K-related pathways showed more limited alterations and MAPK signaling was minimally affected. Detailed descriptions of individual genes have been removed from the main text and are now presented only in the supplementary data.

We have also revised the Discussion section to avoid repetition of the Results. Rather than restating individual gene expression changes, the Discussion now focuses on the biological interpretation and significance of the pathway-level findings. These revisions have improved the clarity, conciseness, and readability of the manuscript.

7. Several grammatical errors and awkward phrases are present.

RESPONSE: We have carefully reviewed the entire manuscript and corrected grammatical errors, typographical mistakes, and awkward phrasing throughout the text. In addition, the manuscript has been thoroughly edited to improve clarity, readability, and consistency of scientific terminology. We have also standardized the use of key terms, particularly those related to cell death mechanisms and immune microenvironment remodeling, to ensure consistent usage throughout the manuscript. We believe these revisions have substantially improved the overall quality and readability of the manuscript.

8. The number of replicates and independent experiments is not always clearly stated. Clarify whether data represent biological or technical replicates.

RESPONSE: We agree that clear reporting of experimental replicates is essential for transparency and reproducibility. In the revised manuscript, we have carefully reviewed the Methods section, figure legends, and Results section to clarify the number and type of replicates used in each experiment. Specifically, we now indicate whether the data represent biological replicates, technical replicates, or both, and we have added the corresponding sample sizes (n values) where appropriate.

For *in vitro* experiments, unless otherwise stated, data were generated from at least three independent biological experiments, with technical replicates performed within each experiment. For animal studies, the number of mice per group is now clearly indicated in both the Methods section and the relevant figure legends. We have also revised the figure legends to explicitly state the number of independent experiments and the nature of the replicates used for statistical analyses. These changes improve the transparency and reproducibility of the study.

9. Ensure all figures include exact n values, statistical tests used and clear labeling of error bars

RESPONSE: In the revised manuscript, we have carefully reviewed and updated all figure legends to ensure that the exact sample size (n value), statistical tests used, and definitions of error bars are clearly stated. Specifically, the number of biological replicates, technical replicates, or animals per group is now provided where applicable. We have also specified the statistical methods used for each analysis, including Student’s t-test, one-way ANOVA, or two-way ANOVA as appropriate. Furthermore, all figure legends now clearly indicate whether the data are presented as mean ± SD or mean ± SEM. These revisions improve the transparency, rigor, and reproducibility of the reported results.

10. Use consistent terminology for pyroptosis-like death and immune micro-environment remodeling.

RESPONSE: We agree that consistent terminology is important for clarity and accurate interpretation of the findings. In the revised manuscript, we have carefully reviewed the text and standardized the terminology used to describe both cell death mechanisms and changes in the tumor immune microenvironment.

Specifically, following the reviewer’s comments regarding the limited evidence for canonical pyroptosis, we have replaced inconsistent terms such as “atypical pyroptosis,” “pyroptosis-like death,” and similar expressions with the more consistent terminology “pyroptosis-associated features” or “pyroptosis-related signaling changes,” where appropriate. In addition, because apoptosis appears to be the predominant mechanism supported by our data, we have revised the text to clearly distinguish apoptosis-related findings from pyroptosis-associated observations.

Similarly, terminology describing immune changes within tumors has been standardized throughout the manuscript. Terms such as “immune micro-environment remodeling,” “immune regulation,” and “immune alteration” have been harmonized using consistent expressions including “tumor immune microenvironment remodeling” and “immune cell infiltration,” depending on the specific context.

**Reviewer #2**

1. This is a well-conducted and clinically relevant study that raises one of the most encountered problems in GBM: Resistance to all treatment modalities, including immune checkpoint inhibitors and CAR-T cell therapies. The authors investigate two host-defense peptides, caerin 1.1 and 1.9 (F1/F3), in U87 and U118 glioblastoma cells and propose ARHGAP26–β-catenin signaling as a mechanistic axis that links peptide treatment to the suppression of pro-invasive effectors, including MMP2, MMP7, and VEGFA. In immunodeficient hosts, F1/F3 has no measurable effect on tumor growth, but in the humanized group, it inhibits U87 growth, increases infiltration of CD8⁺ T cells, promotes M1 polarization, and reduces tumor-cell PD-L1. The paper is a meaningful addition to the field, and I recommend minor revisions.

RESPONSE: We sincerely thank the reviewer for the positive evaluation of our work and for recognizing the clinical relevance of this study. We are encouraged that the reviewer considers our findings a meaningful contribution to the field of glioblastoma research, particularly in the context of overcoming resistance to current immunotherapeutic approaches.

2. The study design is appropriate. My main comment concerns about the interpretation of the humanized NSG model, which is the most important point. The reconstituted human T cells in this system are naive, they have not been chronically stimulated, and lack the exhausted phenotype that defines tumor-infiltrating lymphocytes in real GBM patients. It would be worth adding a brief paragraph discussing whether there is a feasible way to introduce a naive immune compartment into human GBM patients, whether prior literature has addressed this question, or at least acknowledging it as a limitation. The analyses are appropriate, consistent variance reporting, and standard significance thresholds. The supplementary tables and figures are available, The manuscript is well written.
Overall the central finding is well-supported and contributes meaningfully to a field where most candidate therapies fail. The most important revision is the framing of the humanized model.

RESPONSE: We sincerely thank the reviewer for highlighting this important limitation of the PBMC-humanized NSG model. We agree that the human T cells reconstituted in this model are predominantly naïve and have not undergone the chronic antigen stimulation, exhaustion, and functional adaptation that characterize tumor-infiltrating lymphocytes in patients with glioblastoma. Therefore, this model does not fully recapitulate the complex immune landscape of human GBM.

Our intention in using the PBMC-humanized NSG model was to provide an initial assessment of whether the antitumor activity of F1/F3 is influenced by the presence of human immune cells. We agree that the observed increase in CD8⁺ T-cell infiltration should be interpreted within the limitations of this model and may not fully reflect the behavior of exhausted or dysfunctional T cells present in clinical GBM.

In response to the reviewer’s suggestion, we have added a paragraph to the Discussion acknowledging this limitation and discussing the implications for clinical translation. Specifically, we note that while the humanized NSG model provides a useful platform for evaluating interactions between tumor cells and human immune cells, it does not reproduce the chronic immunosuppressive environment of GBM patients. We also discuss that therapeutic strategies aimed at introducing or restoring functional antitumor T-cell populations in GBM patients, including adoptive T-cell therapies, CAR-T cells, tumor-infiltrating lymphocyte (TIL) therapy, and immune checkpoint blockade, have been actively investigated in the literature, although their efficacy remains limited by the highly immunosuppressive tumor microenvironment.

Accordingly, we have revised the manuscript to frame the humanized mouse data more cautiously and to emphasize that the findings provide preliminary evidence that F1/F3 may modulate human immune cell infiltration, rather than fully recapitulating the immune responses occurring in patients with glioblastoma. We agree that future studies using more advanced humanized models and functional characterization of infiltrating immune cells will be important for validating the translational relevance of these observations. The Abstract and Conclusion sections have been modified accordingly.

Thanks very much for reviewing our manuscript, please let me know if further improvement is needed.

Sincerely,

Guoying Ni, PhD
