# Supplementary material for: Caerin 1.1 and 1.9 inhibit glioblastoma growth associated with modulation of the ARHGAP26-β-catenin axis and enhancing intratumoral CD8+ T cell infiltration
Source: PLoS One. 2026 Jul 9;21(7):e0353182. doi: 10.1371/journal.pone.0353182 (PMC13349103; doi:10.1371/journal.pone.0353182)
Supplement: Peer review history

# Peer review: Decision Letter 0

**DOI:** 10.1371/journal.pone.0353182.r001

**Authors:** Ryota Tamura

13 May 2026

PONE-D-26-13623

Caerin 1.1 and 1.9 inhibit glioblastoma growth by regulating the ARHGAP26-β-catenin axis and enhancing intratumoral CD8+ T cell infiltration

PLOS One

Dear Dr. Wang,

Thank you for submitting your manuscript to PLOS ONE. After careful consideration, we feel that it has merit but does not fully meet PLOS ONE’s publication criteria as it currently stands. Therefore, we invite you to submit a revised version of the manuscript that addresses the points raised during the review process.

Please submit your revised manuscript by Jun 27 2026 11:59PM. If you will need more time than this to complete your revisions, please reply to this message or contact the journal office at plosone@plos.org. When you're ready to submit your revision, log on to https://www.editorialmanager.com/pone/ and select the 'Submissions Needing Revision' folder to locate your manuscript file.

Please include the following items when submitting your revised manuscript:

- A letter that responds to each point raised by the academic editor and reviewer(s). You should upload this letter as a separate file labeled 'Response to Reviewers'.
- A marked-up copy of your manuscript that highlights changes made to the original version. You should upload this as a separate file labeled 'Revised Manuscript with Track Changes'.
- An unmarked version of your revised paper without tracked changes. You should upload this as a separate file labeled 'Manuscript'.

If you would like to make changes to your financial disclosure, please include your updated statement in your cover letter. Guidelines for resubmitting your figure files are available below the reviewer comments at the end of this letter.

If applicable, we recommend that you deposit your laboratory protocols in protocols.io to enhance the reproducibility of your results. Protocols.io assigns your protocol its own identifier (DOI) so that it can be cited independently in the future. For instructions see: https://journals.plos.org/plosone/s/submission-guidelines#loc-laboratory-protocols. Additionally, PLOS ONE offers an option for publishing peer-reviewed Lab Protocol articles, which describe protocols hosted on protocols.io. Read more information on sharing protocols at https://plos.org/protocols?utm_medium=editorial-email&utm_source=authorletters&utm_campaign=protocols.

As the corresponding author, your ORCID iD is verified in the submission system and will appear in the published article. PLOS supports the use of ORCID, and we encourage all coauthors to register for an ORCID iD and use it as well. Please encourage your coauthors to verify their ORCID iD within the submission system before final acceptance, as unverified ORCID iDs will not appear in the published article. *Only*  the individual author can complete the verification step; PLOS staff *cannot*  verify ORCID iDs on behalf of authors.

We look forward to receiving your revised manuscript.

Kind regards,

Ryota Tamura

Academic Editor

PLOS One

Journal Requirements:

When submitting your revision, we need you to address these additional requirements.

1.Please ensure that your manuscript meets PLOS ONE's style requirements, including those for file naming. The PLOS ONE style templates can be found at

https://journals.plos.org/plosone/s/file?id=wjVg/PLOSOne_formatting_sample_main_body.pdf and

https://journals.plos.org/plosone/s/file?id=ba62/PLOSOne_formatting_sample_title_authors_affiliations.pdf

2. To comply with PLOS One submissions requirements, in your Methods section, please provide additional information regarding the experiments involving animals and ensure you have included details on (1) methods of anesthesia and/or analgesia, and (2) efforts to alleviate suffering.

3. Thank you for stating the following in the Competing Interests section:

“The authors have declared that no competing interests exist.”

We note that one or more of the authors are employed by a commercial company: Zhongao Biomedical Technology (Guangdong) Co., Ltd., Zhongshan 528400, China

1.        Please provide an amended Funding Statement declaring this commercial affiliation, as well as a statement regarding the Role of Funders in your study. If the funding organization did not play a role in the study design, data collection and analysis, decision to publish, or preparation of the manuscript and only provided financial support in the form of authors' salaries and/or research materials, please review your statements relating to the author contributions, and ensure you have specifically and accurately indicated the role(s) that these authors had in your study. You can update author roles in the Author Contributions section of the online submission form.

Please also include the following statement within your amended Funding Statement.

“The funder provided support in the form of salaries for authors [insert relevant initials], but did not have any additional role in the study design, data collection and analysis, decision to publish, or preparation of the manuscript. The specific roles of these authors are articulated in the ‘author contributions’ section.”

If your commercial affiliation did play a role in your study, please state and explain this role within your updated Funding Statement.

2. Please also provide an updated Competing Interests Statement declaring this commercial affiliation along with any other relevant declarations relating to employment, consultancy, patents, products in development, or marketed products, etc.

Within your Competing Interests Statement, please confirm that this commercial affiliation does not alter your adherence to all PLOS ONE policies on sharing data and materials by including the following statement: "This does not alter our adherence to  PLOS ONE policies on sharing data and materials.” (as detailed online in our guide for authors http://journals.plos.org/plosone/s/competing-interests) . If this adherence statement is not accurate and  there are restrictions on sharing of data and/or materials, please state these. Please note that we cannot proceed with consideration of your article until this information has been declared.

Please include both an updated Funding Statement and Competing Interests Statement in your cover letter. We will change the online submission form on your behalf.

4. PLOS ONE now requires that authors provide the original uncropped and unadjusted images underlying all blot or gel results reported in a submission’s figures or Supporting Information files. This policy and the journal’s other requirements for blot/gel reporting and figure preparation are described in detail at https://journals.plos.org/plosone/s/figures#loc-blot-and-gel-reporting-requirements and https://journals.plos.org/plosone/s/figures#loc-preparing-figures-from-image-files. When you submit your revised manuscript, please ensure that your figures adhere fully to these guidelines and provide the original underlying images for all blot or gel data reported in your submission. See the following link for instructions on providing the original image data: https://journals.plos.org/plosone/s/figures#loc-original-images-for-blots-and-gels.

In your cover letter, please note whether your blot/gel image data are in Supporting Information or posted at a public data repository, provide the repository URL if relevant, and provide specific details as to which raw blot/gel images, if any, are not available. Email us at plosone@plos.org if you have any questions.

5. We note that your Data Availability Statement is currently as follows: All relevant data are within the manuscript and in Supporting Information files.

Please confirm at this time whether or not your submission contains all raw data required to replicate the results of your study. Authors must share the “minimal data set” for their submission. PLOS defines the minimal data set to consist of the data required to replicate all study findings reported in the article, as well as related metadata and methods (https://journals.plos.org/plosone/s/data-availability#loc-minimal-data-set-definition).

For example, authors should submit the following data:

- The values behind the means, standard deviations and other measures reported;

- The values used to build graphs;

- The points extracted from images for analysis.

Authors do not need to submit their entire data set if only a portion of the data was used in the reported study.

If your submission does not contain these data, please either upload them as Supporting Information files or deposit them to a stable, public repository and provide us with the relevant URLs, DOIs, or accession numbers. For a list of recommended repositories, please see https://journals.plos.org/plosone/s/recommended-repositories.

If there are ethical or legal restrictions on sharing a de-identified data set, please explain them in detail (e.g., data contain potentially sensitive information, data are owned by a third-party organization, etc.) and who has imposed them (e.g., an ethics committee). Please also provide contact information for a data access committee, ethics committee, or other institutional body to which data requests may be sent. If data are owned by a third party, please indicate how others may request data access.

6. If the reviewer comments include a recommendation to cite specific previously published works, please review and evaluate these publications to determine whether they are relevant and should be cited. There is no requirement to cite these works unless the editor has indicated otherwise.

[Note: HTML markup is below. Please do not edit.]

Reviewers' comments:

Reviewer's Responses to Questions

**Comments to the Author**

1. Is the manuscript technically sound, and do the data support the conclusions?

The manuscript must describe a technically sound piece of scientific research with data that supports the conclusions. Experiments must have been conducted rigorously, with appropriate controls, replication, and sample sizes. The conclusions must be drawn appropriately based on the data presented.

Reviewer #1: Partly

Reviewer #2: Yes

**********

2. Has the statistical analysis been performed appropriately and rigorously?

Reviewer #1: No

Reviewer #2: Yes

**********

3. Have the authors made all data underlying the findings in their manuscript fully available?

The PLOS Data policy requires authors to make all data underlying the findings described in their manuscript fully available without restriction, with rare exception (please refer to the Data Availability Statement in the manuscript PDF file). The data should be provided as part of the manuscript or its supporting information, or deposited to a public repository. For example, in addition to summary statistics, the data points behind means, medians and variance measures should be available. If there are restrictions on publicly sharing data—e.g. participant privacy or use of data from a third party—those must be specified.

Reviewer #1: Yes

Reviewer #2: Yes

**********

4. Is the manuscript presented in an intelligible fashion and written in standard English?

PLOS ONE does not copyedit accepted manuscripts, so the language in submitted articles must be clear, correct, and unambiguous. Any typographical or grammatical errors should be corrected at revision, so please note any specific errors here.

Reviewer #1: Yes

Reviewer #2: Yes

**********

5. Review Comments to the Author

Please use the space provided to explain your answers to the questions above. You may also include additional comments for the author, including concerns about dual publication, research ethics, or publication ethics. (Please upload your review as an attachment if it exceeds 20,000 characters)

Reviewer #1: This manuscript titled, “Caerin 1.1 and 1.9 inhibit glioblastoma growth by regulating the ARHGAP26–β-catenin axis and enhancing intratumoral CD8⁺ T cell infiltration” investigates the anti-tumor effects of caerin peptides (1.1 and 1.9) in glioblastoma using in vitro assays, xenograft models, and a PBMC-humanized mouse model. The study addresses an important clinical challenge in glioblastoma and presents a multi-layered experimental approach. The integration of tumor-intrinsic signaling and immune modulation is a strength. However, several issues regarding mechanistic rigor, data interpretation, redundancy, and clarity need to be considered before the manuscript can be considered for publication.

Major Comments:

1.The central claim that ARHGAP26 mediates the anti-tumor effects is not fully supported. The study shows correlation, however, no causal experiments are conducted. There is no knockdown or overexpression validation of ARHGAP26.

2.The manuscript concludes that caerin peptides induce “atypical pyroptosis.” However, IL-1β and IL-18 secretion is minimal or absent, Key pyroptosis effectors (e.g., GSDMD) are downregulated and no direct evidence of inflammasome activation is provided. Hence, The data do not convincingly support pyroptosis as a major mechanism.

3.The manuscript states that apoptosis is the primary mechanism of cell death based on gene expression data. But there is no functional validation like caspase inhibitor assay or annexinV/PI quantification. Authors should perform additional validation experiments to confirm the mechanism of cell death

4.The PBMC-humanized mouse experiments use very small group sizes (n=3). This limits statistical power, weakens conclusions about immune mechanisms. Authors should increase the sample size or explicitly acknowledge this limitation in the Discussion.

5.The study shows increased CD8⁺ T cell infiltration but does not assess their functional state. Increased infiltration does not necessarily imply enhanced anti-tumor activity. Authors should include functional assays to support their claim

Minor Concerns

1.The description of PCR array results is excessively long and appears twice (Results and Discussion). Summarize key findings concisely in the main text.

2.Several grammatical errors and awkward phrases are present.

3.The number of replicates and independent experiments is not always clearly stated. Clarify whether data represent biological or technical replicates.

4.Ensure all figures include exact n values, statistical tests used and clear labeling of error bars

5.Use consistent terminology for pyroptosis-like death and immune micro-environment remodeling

Reviewer #2: This is a well-conducted and clinically relevant study that raises one of the most encountered problems in GBM: Resistance to all treatment modalities, including immune checkpoint inhibitors and CAR-T cell therapies. The authors investigate two host-defense peptides, caerin 1.1 and 1.9 (F1/F3), in U87 and U118 glioblastoma cells and propose ARHGAP26–β-catenin signaling as a mechanistic axis that links peptide treatment to the suppression of pro-invasive effectors, including MMP2, MMP7, and VEGFA. In immunodeficient hosts, F1/F3 has no measurable effect on tumor growth, but in the humanized group, it inhibits U87 growth, increases infiltration of CD8⁺ T cells, promotes M1 polarization, and reduces tumor-cell PD-L1. The paper is a meaningful addition to the field, and I recommend minor revisions.

The study design is appropriate. My main comment concerns about the interpretation of the humanized NSG model, which is the most important point. The reconstituted human T cells in this system are naive, they have not been chronically stimulated, and lack the exhausted phenotype that defines tumor-infiltrating lymphocytes in real GBM patients. It would be worth adding a brief paragraph discussing whether there is a feasible way to introduce a naive immune compartment into human GBM patients, whether prior literature has addressed this question, or at least acknowledging it as a limitation. The analyses are appropriate, consistent variance reporting, and standard significance thresholds. The supplementary tables and figures are available, The manuscript is well written.

Overall the central finding is well-supported and contributes meaningfully to a field where most candidate therapies fail. The most important revision is the framing of the humanized model.

**********

6. PLOS authors have the option to publish the peer review history of their article (what does this mean?). If published, this will include your full peer review and any attached files.

If you choose “no”, your identity will remain anonymous but your review may still be made public.

**Do you want your identity to be public for this peer review?**  For information about this choice, including consent withdrawal, please see our Privacy Policy.

Reviewer #1: No

Reviewer #2: No

**********

[NOTE: If reviewer comments were submitted as an attachment file, they will be attached to this email and accessible via the submission site. Please log into your account, locate the manuscript record, and check for the action link "View Attachments". If this link does not appear, there are no attachment files.]

To ensure your figures meet our technical requirements, please review our figure guidelines: https://journals.plos.org/plosone/s/figures

You may also use PLOS’s free figure tool, NAAS, to help you prepare publication quality figures: https://journals.plos.org/plosone/s/figures#loc-tools-for-figure-preparation.

NAAS will assess whether your figures meet our technical requirements by comparing each figure against our figure specifications.

Attachment Submitted filename: Comments_PlosONE.docx

# Author comment: Author response to Decision Letter 1

**DOI:** 10.1371/journal.pone.0353182.r002

5 Jun 2026

Dear Editor and Reviewers,

We sincerely thank you for your careful evaluation of our manuscript and for the constructive and insightful comments provided. We have carefully considered all suggestions and revised the manuscript accordingly to improve its clarity, scientific rigor, and overall presentation. In the revised manuscript, all modifications have been highlighted in red fonts, and detailed point-by-point responses to each comment are provided below.

Editor

1. Please ensure that your manuscript meets PLOS ONE's style requirements, including those for file naming.

RESPONSE: We have carefully reviewed the manuscript and revised the formatting, file naming, and presentation to ensure compliance with all PLOS ONE style requirements.

2. To comply with PLOS One submissions requirements, in your Methods section, please provide additional information regarding the experiments involving animals and ensure you have included details on (1) methods of anesthesia and/or analgesia, and (2) efforts to alleviate suffering.

RESPONSE: We have revised the Methods section to provide additional details regarding the animal experiments, including the methods of anesthesia and euthanasia, humane endpoint criteria, daily monitoring procedures, and measures taken to minimize animal suffering and distress throughout the study. These additions were incorporated to ensure compliance with PLOS ONE animal research reporting requirements and ARRIVE guidelines.

3. Please provide an amended Funding Statement declaring this commercial affiliation, as well as a statement regarding the Role of Funders in your study. If the funding organization did not play a role in the study design, data collection and analysis, decision to publish, or preparation of the manuscript and only provided financial support in the form of authors' salaries and/or research materials, please review your statements relating to the author contributions, and ensure you have specifically and accurately indicated the role(s) that these authors had in your study. You can update author roles in the Author Contributions section of the online submission form. Please also include the following statement within your amended Funding Statement.

RESPONSE: We have amended the Funding Statement to clarify the commercial affiliation and the role of the funder in this study. Specifically, we added the following statement: “The funder provided support in the form of salaries for authors [JL, YL, QF, XL, GN], but did not have any additional role in the study design, data collection and analysis, decision to publish, or preparation of the manuscript. The specific roles of these authors are articulated in the ‘Author Contributions’ section.” We have also reviewed and updated the Author Contributions section in the online submission system to ensure that the contributions of all authors are accurately described

4. Please also provide an updated Competing Interests Statement declaring this commercial affiliation along with any other relevant declarations relating to employment, consultancy, patents, products in development, or marketed products, etc. Within your Competing Interests Statement, please confirm that this commercial affiliation does not alter your adherence to all PLOS ONE policies on sharing data and materials by including the following statement: "This does not alter our adherence to PLOS ONE policies on sharing data and materials.”

RESPONSE: We have updated the Competing Interests Statement to declare the relevant commercial affiliation and related author associations. We also confirmed that these affiliations do not influence compliance with the journal’s policies on data and material sharing. Specifically, we included the statement: “This does not alter our adherence to PLOS ONE policies on sharing data and materials.” In addition, we carefully reviewed the manuscript to ensure that all relevant declarations regarding employment, affiliations, and potential competing interests were appropriately disclosed。

5. PLOS ONE now requires that authors provide the original uncropped and unadjusted images underlying all blot or gel results reported in a submission’s figures or Supporting Information files. In your cover letter, please note whether your blot/gel image data are in Supporting Information or posted at a public data repository, provide the repository URL if relevant, and provide specific details as to which raw blot/gel images, if any, are not available.

RESPONSE: We have provided the original uncropped and unadjusted blot/gel images underlying the relevant figures as Supporting Information files in accordance with PLOS ONE requirements. We have also updated the cover letter to indicate that the raw blot/gel image data are included in the Supporting Information S1 File. All available raw images have been provided, and no relevant blot/gel image data have been intentionally omitted.

6. We note that your Data Availability Statement is currently as follows: All relevant data are within the manuscript and in Supporting Information files. Please confirm at this time whether or not your submission contains all raw data required to replicate the results of your study.

RESPONSE: We confirm that the submission contains all raw data required to replicate the findings of this study. All relevant experimental data, including raw blot/gel images and supporting datasets, have been provided within the manuscript and Supporting Information files in accordance with PLOS ONE data availability requirements.

Reviewer #1:

1. The central claim that ARHGAP26 mediates the anti-tumor effects is not fully supported. The study shows correlation, however, no causal experiments are conducted. There is no knockdown or overexpression validation of ARHGAP26.

RESPONSE: We thank the reviewer for this important comment. We agree that the current study primarily demonstrates an association between F1/F3 treatment and modulation of the ARHGAP26–β-catenin axis, rather than definitive causal evidence. In the present work, ARHGAP26 was identified through our proteomic profiling and subsequently validated by western blotting together with downstream β-catenin-related proteins, including MMP2, MMP7, and VEGFA. These findings suggest that ARHGAP26 is likely involved in the antitumor effects of F1/F3 in glioblastoma cells.

However, we acknowledge that direct mechanistic validation using ARHGAP26 knockdown or overexpression experiments would further strengthen the causal interpretation. Due to time limitations and the extensive additional experiments required, these studies were beyond the scope of the current manuscript. We have therefore revised the manuscript to moderate the mechanistic claims and clarify that our data support a strong association between F1/F3 treatment and regulation of the ARHGAP26–β-catenin signaling pathway, rather than definitive proof of causality. We have also added this limitation in the Discussion section and highlighted ARHGAP26 functional validation as an important direction for future studies.

In addition, to avoid overstating mechanistic causality, we revised the manuscript title and related text throughout the manuscript to reflect that the observed changes in the ARHGAP26–β-catenin axis are associated with, rather than definitively proven to mediate, the antitumor effects of F1/F3.

2. The manuscript concludes that caerin peptides induce “atypical pyroptosis.” However, IL-1β and IL-18 secretion is minimal or absent, Key pyroptosis effectors (e.g., GSDMD) are downregulated and no direct evidence of inflammasome activation is provided. Hence, the data do not convincingly support pyroptosis as a major mechanism.

RESPONSE: We agree that the current data do not provide sufficient evidence to support canonical inflammasome-mediated pyroptosis as a major mechanism of F1/F3-induced cell death in glioblastoma cells. Our original interpretation was based on the substantial LDH release and membrane damage observed following F1/F3 treatment, together with the differential expression of several pyroptosis-related genes identified by PCR array analysis. However, as the reviewer correctly noted, IL-1β and IL-18 secretion was minimal or undetectable, GSDMD expression was downregulated, and no direct evidence of inflammasome activation or gasdermin cleavage was provided.

We therefore agree that the evidence does not support the conclusion that pyroptosis is a dominant mechanism underlying F1/F3-mediated cytotoxicity in U87 and U118 cells. In the revised manuscript, we have moderated our interpretation throughout the Results, Discussion, figure legends, and section headings. Specifically, we replaced the term “atypical pyroptosis” with more cautious wording such as “pyroptosis-associated features” or “pyroptosis-related signaling changes” where appropriate. We also revised the Results section to emphasize that apoptosis-related pathways exhibited the most extensive transcriptional changes and likely represent the predominant mechanism of cell death induced by F1/F3.

Furthermore, we have added a statement in the Discussion acknowledging that the absence of robust IL-1β/IL-18 secretion, the downregulation of canonical pyroptosis effectors, and the lack of direct inflammasome activation assays limit our ability to conclude that pyroptosis contributes substantially to the observed antitumor effects. Future studies examining gasdermin cleavage, inflammasome activation, and caspase-dependent signaling will be required to clarify the contribution of pyroptosis-related pathways to F1/F3-induced glioblastoma cell death.

Accordingly, the manuscript now presents apoptosis as the principal mechanism associated with F1/F3-induced cytotoxicity, while pyroptosis-related signaling changes are discussed as secondary observations that warrant further investigation.

3. The manuscript states that apoptosis is the primary mechanism of cell death based on gene expression data. But there is no functional validation like caspase inhibitor assay or annexinV/PI quantification. Authors should perform additional validation experiments to confirm the mechanism of cell death.

RESPONSE: We thank the reviewer for this valuable comment and agree that direct functional validation would strengthen the conclusion that apoptosis is the predominant mechanism of F1/F3-induced cell death in glioblastoma cells. In the current study, our conclusion was based on several lines of evidence, including the extensive transcriptional changes observed in apoptosis-related genes, the upregulation of both intrinsic and extrinsic apoptosis-associated pathways, and the lack of evidence supporting ferroptosis or necroptosis based on inhibitor studies. In addition, our previous studies demonstrated that F1/F3 induces caspase-3-dependent apoptosis in multiple cancer cell types, including HeLa cells and melanoma cells, providing supporting evidence for a similar mechanism in glioblastoma cells.

However, we agree that the present study does not include direct functional assays such as Annexin V/PI staining, caspase inhibition experiments, or measurement of caspase activation in U87 and U118 cells. Therefore, we have revised the manuscript to moderate our interpretation and avoid presenting apoptosis as definitively proven. Specifically, we now state that the data are consistent with apoptosis being the predominant cell death pathway rather than conclusively demonstrating it. We have also revised the Discussion section to acknowledge the lack of direct functional validation as a limitation of the current study.

We agree that additional experiments, including Annexin V/PI analysis, caspase activation assays, and caspase inhibition studies, would provide stronger mechanistic evidence and will be pursued in future investigations to further define the cell death pathways induced by F1/F3 in glioblastoma cells. "Our interpretation is also supported by our recent study demonstrating that F1/F3 induces caspase-3 activation and apoptosis-associated cell death in epithelial cancer cells (Luo et al., Sci Rep, 2025)."

4. The PBMC-humanized mouse experiments use very small group sizes (n = 3). This limits statistical power, weakens conclusions about immune mechanisms. Authors should increase the sample size or explicitly acknowledge this limitation in the Discussion.

RESPONSE: We agree that the small sample size in the PBMC-humanized mouse experiment (n = 3 per group) limits the statistical power of the study and reduces the strength of conclusions regarding the underlying immune mechanisms. The humanized NSG mouse model is technically demanding, time-consuming, and costly, particularly when establishing human immune cell engraftment and maintaining animals over an extended experimental period. Consequently, the current experiment was designed as a proof-of-concept study to evaluate whether F1/F3 could exert antitumor effects in the presence of a human immune system.

Despite the limited sample size, we observed consistent effects across multiple independent endpoints, including reduced tumor growth, increased infiltration of human CD8⁺ T cells and macrophages, and reduced PD-L1 expression within the tumor microenvironment. Nevertheless, we agree that these findings should be interpreted with caution.

We have therefore revised the Discussion section to explicitly acknowledge the limited sample size as a study limitation. Specifically, we note that the results provide preliminary evidence supporting the immunomodulatory activity of F1/F3 in a humanized glioblastoma model, but that larger studies with increased animal numbers and additional mechanistic analyses will be required to confirm these observations and more rigorously define the immune pathways involved.

In the revised manuscript, we have also moderated the language describing the immune mechanisms to avoid overinterpretation of findings derived from this exploratory study.

5. The study shows increased CD8⁺ T cell infiltration but does not assess their functional state. Increased infiltration does not necessarily imply enhanced anti-tumor activity. Authors should include functional assays to support their claim.

RESPONSE: We agree that increased CD8⁺ T-cell infiltration alone does not necessarily indicate enhanced antitumor function. Functional characterization of infiltrating CD8⁺ T cells, such as assessment of activation markers, cytokine production (e.g., IFN-γ, TNF-α), cytotoxic molecules (e.g., granzyme B, perforin), or ex vivo killing assays, would provide stronger evidence for enhanced antitumor activity.

In the current study, our primary objective was to determine whether F1/F3 could alter the immune landscape within the glioblastoma tumor microenvironment. We found that F1/F3 treatment was associated with increased infiltration of human CD8⁺ T cells, reduced PD-L1 expression, and significant suppression of tumor growth in the humanized NSG mouse model. While these observations are consistent with enhanced antitumor immunity, we agree that they do not directly demonstrate improved CD8⁺ T-cell functionality.

Due to limitations in sample availability and the exploratory nature of the humanized mouse experiments, functional analyses of tumor-infiltrating CD8⁺ T cells were not performed in the current study. We have therefore revised the manuscript to avoid implying that increased infiltration necessarily reflects enhanced effector function. Specifically, we now state that F1/F3 is associated with increased CD8⁺ T-cell infiltration rather than enhanced CD8⁺ T-cell activity.

In addition, we have added a statement in the Discussion acknowledging this limitation and highlighting future studies aimed at characterizing the activation, exhaustion, and cytotoxic status of tumor-infiltrating CD8⁺ T cells. Such experiments will be important for defining the mechanisms by which F1/F3 modulates antitumor immunity in glioblastoma.

6. The description of PCR array results is excessively long and appears twice (Results and Discussion). Summarize key findings concisely in the main text.

RESPONSE: We agree that the original description of the PCR array results was overly detailed and repetitive. In response, we have substantially condensed the presentation of the PCR array data in both the Results and Dis

Attachment Submitted filename: Response to reviewer.docx

# Peer review: Decision Letter 1

**DOI:** 10.1371/journal.pone.0353182.r003

**Authors:** Ryota Tamura

21 Jun 2026

Caerin 1.1 and 1.9 inhibit glioblastoma growth associated with modulation of the ARHGAP26-β-catenin axis and enhancing intratumoral CD8+

T cell infiltration

PONE-D-26-13623R1

Dear Dr. Wang,

We’re pleased to inform you that your manuscript has been judged scientifically suitable for publication and will be formally accepted for publication once it meets all outstanding technical requirements.

Within one week, you’ll receive an e-mail detailing the required amendments. When these have been addressed, you’ll receive a formal acceptance letter and your manuscript will be scheduled for publication.

An invoice will be generated when your article is formally accepted. Please note, if your institution has a publishing partnership with PLOS and your article meets the relevant criteria, all or part of your publication costs will be covered. Please make sure your user information is up-to-date by logging into Editorial Manager at Editorial Manager® and clicking the ‘Update My Information' link at the top of the page. For questions related to billing, please contact billing support.

If your institution or institutions have a press office, please notify them about your upcoming paper to help maximize its impact. If they’ll be preparing press materials, please inform our press team as soon as possible -- no later than 48 hours after receiving the formal acceptance. Your manuscript will remain under strict press embargo until 2 pm Eastern Time on the date of publication. For more information, please contact onepress@plos.org.

Kind regards,

Ryota Tamura

Academic Editor

PLOS One

Additional Editor Comments (optional):

This manuscript is in a nice condition. Thank you very much for your effort.

Reviewers' comments:

Reviewer's Responses to Questions

**Comments to the Author**

1. If the authors have adequately addressed your comments raised in a previous round of review and you feel that this manuscript is now acceptable for publication, you may indicate that here to bypass the “Comments to the Author” section, enter your conflict of interest statement in the “Confidential to Editor” section, and submit your "Accept" recommendation.

Reviewer #1: All comments have been addressed

Reviewer #2: All comments have been addressed

**********

2. Is the manuscript technically sound, and do the data support the conclusions?

The manuscript must describe a technically sound piece of scientific research with data that supports the conclusions. Experiments must have been conducted rigorously, with appropriate controls, replication, and sample sizes. The conclusions must be drawn appropriately based on the data presented.

Reviewer #1: Yes

Reviewer #2: Yes

**********

3. Has the statistical analysis been performed appropriately and rigorously?

Reviewer #1: Yes

Reviewer #2: Yes

**********

4. Have the authors made all data underlying the findings in their manuscript fully available?

The PLOS Data policy requires authors to make all data underlying the findings described in their manuscript fully available without restriction, with rare exception (please refer to the Data Availability Statement in the manuscript PDF file). The data should be provided as part of the manuscript or its supporting information, or deposited to a public repository. For example, in addition to summary statistics, the data points behind means, medians and variance measures should be available. If there are restrictions on publicly sharing data—e.g. participant privacy or use of data from a third party—those must be specified.

Reviewer #1: Yes

Reviewer #2: Yes

**********

5. Is the manuscript presented in an intelligible fashion and written in standard English?

PLOS ONE does not copyedit accepted manuscripts, so the language in submitted articles must be clear, correct, and unambiguous. Any typographical or grammatical errors should be corrected at revision, so please note any specific errors here.

Reviewer #1: Yes

Reviewer #2: Yes

**********

6. Review Comments to the Author

Please use the space provided to explain your answers to the questions above. You may also include additional comments for the author, including concerns about dual publication, research ethics, or publication ethics. (Please upload your review as an attachment if it exceeds 20,000 characters)

Reviewer #1: Manuscript has been revised appropriately and all the shortcomings have been addressed. It can be accepted for publication

Reviewer #2: The authors have satisfactorily addressed all the comments and recommendations raised in the previous review. The revised manuscript reflects the requested corrections, and I have no further concerns regarding the methodology, results, or interpretation presented. I recommend acceptance of the manuscript in its current form.

**********

7. PLOS authors have the option to publish the peer review history of their article (what does this mean?). If published, this will include your full peer review and any attached files.

If you choose “no”, your identity will remain anonymous but your review may still be made public.

**Do you want your identity to be public for this peer review?**  For information about this choice, including consent withdrawal, please see our Privacy Policy.

Reviewer #1: No

Reviewer #2: No

**********

# Editor report: Acceptance letter

**DOI:** 10.1371/journal.pone.0353182.r004

**Authors:** Ryota Tamura

PONE-D-26-13623R1

PLOS One

Dear Dr. Wang,

I'm pleased to inform you that your manuscript has been deemed suitable for publication in PLOS One. Congratulations! Your manuscript is now being handed over to our production team.

At this stage, our production department will prepare your paper for publication. This includes ensuring the following:

* All references, tables, and figures are properly cited

* All relevant supporting information is included in the manuscript submission,

* There are no issues that prevent the paper from being properly typeset

You will receive further instructions from the production team, including instructions on how to review your proof when it is ready. Please keep in mind that we are working through a large volume of accepted articles, so please give us a few days to review your paper and let you know the next and final steps.

Lastly, if your institution or institutions have a press office, please let them know about your upcoming paper now to help maximize its impact. If they'll be preparing press materials, please inform our press team within the next 48 hours. Your manuscript will remain under strict press embargo until 2 pm Eastern Time on the date of publication. For more information, please contact onepress@plos.org.

You will receive an invoice from PLOS for your publication fee after your manuscript has reached the completed accept phase. If you receive an email requesting payment before acceptance or for any other service, this may be a phishing scheme. Learn how to identify phishing emails and protect your accounts at https://explore.plos.org/phishing.

If we can help with anything else, please email us at customercare@plos.org.

Thank you for submitting your work to PLOS ONE and supporting open access.

Kind regards,

PLOS ONE Editorial Office Staff

on behalf of

Dr. Ryota Tamura

Academic Editor

PLOS One
